# CAPLA: improved prediction of protein–ligand binding affinity by a deep learning approach based on a cross-attention mechanism

**DOI:** 10.1093/bioinformatics/btad049

**Published:** 2023-01-23

**Authors:** Zhi Jin, Tingfang Wu, Taoning Chen, Deng Pan, Xuejiao Wang, Jingxin Xie, Lijun Quan, Qiang Lyu

**Affiliations:** School of Computer Science and Technology, Soochow University, Suzhou 215006, China; School of Computer Science and Technology, Soochow University, Suzhou 215006, China; Province Key Lab for Information Processing Technologies, Soochow University, Suzhou 215006, China; Collaborative Innovation Center of Novel Software Technology and Industrialization, Nanjing 210000, China; School of Computer Science and Technology, Soochow University, Suzhou 215006, China; School of Computer Science and Technology, Soochow University, Suzhou 215006, China; School of Computer Science and Technology, Soochow University, Suzhou 215006, China; School of Computer Science and Technology, Soochow University, Suzhou 215006, China; School of Computer Science and Technology, Soochow University, Suzhou 215006, China; Province Key Lab for Information Processing Technologies, Soochow University, Suzhou 215006, China; Collaborative Innovation Center of Novel Software Technology and Industrialization, Nanjing 210000, China; School of Computer Science and Technology, Soochow University, Suzhou 215006, China; Province Key Lab for Information Processing Technologies, Soochow University, Suzhou 215006, China; Collaborative Innovation Center of Novel Software Technology and Industrialization, Nanjing 210000, China

## Abstract

**Motivation:**

Accurate and rapid prediction of protein–ligand binding affinity is a great challenge currently encountered in drug discovery. Recent advances have manifested a promising alternative in applying deep learning-based computational approaches for accurately quantifying binding affinity. The structure complementarity between protein-binding pocket and ligand has a great effect on the binding strength between a protein and a ligand, but most of existing deep learning approaches usually extracted the features of pocket and ligand by these two detached modules.

**Results:**

In this work, a new deep learning approach based on the cross-attention mechanism named CAPLA was developed for improved prediction of protein–ligand binding affinity by learning features from sequence-level information of both protein and ligand. Specifically, CAPLA employs the cross-attention mechanism to capture the mutual effect of protein-binding pocket and ligand. We evaluated the performance of our proposed CAPLA on comprehensive benchmarking experiments on binding affinity prediction, demonstrating the superior performance of CAPLA over state-of-the-art baseline approaches. Moreover, we provided the interpretability for CAPLA to uncover critical functional residues that contribute most to the binding affinity through the analysis of the attention scores generated by the cross-attention mechanism. Consequently, these results indicate that CAPLA is an effective approach for binding affinity prediction and may contribute to useful help for further consequent applications.

**Availability and implementation:**

The source code of the method along with trained models is freely available at https://github.com/lennylv/CAPLA.

**Supplementary information:**

Supplementary data are available at *Bioinformatics* online.

## 1 Introduction

The interactions between proteins and small molecule ligands involve in a variety of biological processes, including enzymatic catalysis and signaling transduction. Furthermore, drugs usually act as ligands and interact with target proteins to exert their effects in drug discovery (Clark *et al.*, 2021). The binding affinity is an important characterization of the strength of the interactions between proteins and ligands. As a consequence, accurate determination of protein–ligand binding affinity is of great significance in drug screening and drug repurposing (Stokes *et al.*, 2020).

The determination of protein–ligand binding affinity by experimental methods is generally considered the most reliable (Gapsys *et al.*, 2020; Liu *et al.*, 2007). However, experimental determination of protein–ligand affinity only covers a small portion of protein–ligand pairs, and it is time-consuming and costly in many cases. Consequently, it is desirable to develop alternative computational methods to accurately estimate protein–ligand binding affinity. More specifically, the estimation of binding affinity by computational methods can contribute to prioritizing the appropriate drug candidates from numerous candidates for succedent experimental testing, thereby speeding up the process of drug design (Wang *et al.*, 2022; Zhao *et al.*, 2020).

Benefiting from the development of the computer-aided drug design method, various physics-based computational methods were proposed to accurately estimate protein–ligand binding affinity, including molecular dynamics simulations (Wang *et al.*, 2020) and free energy simulations (Wang *et al.*, 2021a). However, these methods in screening large-scale protein–ligand complexes remain a significant challenge of a huge amount of computational overhead. In contrast, molecular docking methods (Song *et al.*, 2021; Wu *et al.*, 2018) were capable of predicting protein–ligand binding affinity at an affordable computational overhead, but their prediction of binding affinity was not accurate enough. As compared to physics-based methods, a number of traditional machine-learning-based computational methods (Karimi *et al.*, 2019; Liu *et al.*, 2022) were also developed to improve the prediction performance of protein–ligand binding affinity, such as RFscore based on random forest (Ballester and Mitchell, 2010; Wang and Chan, 2022), and Pred-binding based on support vector machine (Shar *et al.*, 2016). However, the performance of such machine-learning-based methods heavily relies on the design of effective manually extracted features, which requires considerable domain knowledge (Chauhan and Singh, 2018).

As deep learning techniques are capable of automatically learning the feature representation from primitive inputs without any domain knowledge (LeCun *et al.*, 2015), they have attracted a substantial amount of attention. Thus far, multifarious deep learning-based methods were already developed to predict protein–ligand binding affinity, and such methods can be divided into two groups according to whether the 3D structure information, named structure- and sequence-based methods. The structure-based methods treated protein–ligand complexes as 3D-grids or molecular graphs, such as Pafnucy (Stepniewska-Dziubinska *et al.*, 2018), OnionNet (Zheng *et al.*, 2019), FAST (Jones *et al.*, 2021) and IGN (Jiang *et al.*, 2021). For example, the recently developed method IGN (Li *et al.*, 2021) employs graph neural networks to learn useful representation from molecular graph representation of complexes to perform the binding affinity prediction task. However, 3D-grid or molecular graph representation for protein–ligand complexes result in huge computation cost in large-scale binding affinity prediction task. Additionally, the performance of these structure-based methods heavily relies on the high-quality 3D structures of complexes, which greatly limit their applications to practical tasks. The sequence-based methods took 1D sequence of proteins and ligands alone as the model inputs, such as DeepDTA (Öztürk *et al.*, 2018) and DeepDTAF (Wang *et al.*, 2021b). For example, DeepDTAF extracted useful information from the protein and pocket sequences as well as the simplified molecular-input line-entry system (SMILES) of ligands to predict binding affinity (Wang *et al.*, 2021b). It has been well established that the structure complementarity between protein-binding pockets and ligands significantly affects the binding strength between a protein and a ligand (Wang *et al.*, 2011). However, existing sequence-based methods usually learned the feature representation of protein-binding pockets and ligands by two detached modules and simply concatenated the extracted feature vectors, rather than taking into account the mutual interaction between them, which is valuable for binding affinity prediction.

In this work, in pursuit of fully considering the mutual interaction between pockets and ligands, we proposed a new deep learning method based on the cross-attention mechanism, referred to as Cross-Attention for Protein-Ligand binding Affinity (CAPLA), for improved prediction of binding affinity using sequence-level information of both protein and ligand only. More specifically, CAPLA contains three inputs, namely the protein and pocket input representations that comprise amino acid types, secondary structure elements (SSEs) and residue physicochemical properties, as well as the ligand SMILES strings. Then, CAPLA leverages the cross-attention mechanism to capture the mutual interaction features between pockets and ligands, and then adopts the dilated convolutions to learn multiscale long-range features for proteins, pockets and ligands, respectively. Finally, the learned feature vectors of proteins, pockets and ligands are concatenated as the input of a fully connected neural network (FNN) to predict the protein–ligand binding affinity. Experiments of protein–ligand binding affinity prediction on two test datasets demonstrated that CAPLA achieves improved performance with comparisons to several state-of-the-art baseline methods. In addition, we also conducted two external independent tests for CAPLA, and the results demonstrated its superior generalization capability. Moreover, we provided the interpretability for CAPLA by analyzing the attention score in each residue within protein-binding pocket sequences, and found that our method can capture critical functional residues that are important contribution to the protein–ligand binding. Based on these results, it can be concluded that CAPLA is an effective approach for protein–ligand affinity prediction and may contribute to useful guidance for further drug development.

## 2 Materials and methods

### 2.1 Datasets

The commonly used dataset of protein–ligand binding affinity was derived from the PDBbind database of version 2016 (Liu *et al.*, 2017). This database was usually segmented into three overlapping subsets, namely the general set, the refined set and the core 2016 set. Specifically, the general set contains all available data, and now a total of 13 285 protein–ligand complexes are included. The refined set is a subset of the general set, which contains 4057 high-quality complexes in total. The core 2016 set comprises 290 complexes by carefully selecting from the refined set, and this set is usually designed as a high-quality benchmark for evaluating protein–ligand binding affinity prediction methods. Additionally, to evaluate CAPLA on the CASF-2013 datset (Li *et al.*, 2014), all protein–ligand complexes in the general set that overlap with the 195 complexes included in CASF-2013 are needed to exclude, so there are 89 overlapping complexes in total removed from the general set. Here, we adopted the same manner in Pafnucy (Stepniewska-Dziubinska *et al.*, 2018) to partition the training and validation sets, i.e. 1000 complexes were randomly selected from the refined set to constitute the validation set, and a total of 11 906 complexes remaining in the general set constituted the training set. The core 2016 set and the CASF-2013 set were used as two benchmark test sets, and we named them Test2016_290 and Test2013_195, respectively. Notably, the Smith–Waterman similarity (Pearson, 1991) between each pair of protein sequences (one from the core 2016 set and the other from the training set) was at most 60% for 99% protein sequence pairs.

To evaluate the generalization capability of our method, we also employed the CSAR-HiQ dataset (Dunbar *et al.*, 2011) as an additional test set, which consists of two subsets containing 176 and 167 protein–ligand complexes, respectively. Because of the overlap between these two subsets and the training set, we excluded the overlapping complexes from both subsets according to PDB IDs, thus obtaining two test sets consisting of 51 and 36 complexes, respectively. Conveniently, we named these two sets CSAR-HiQ_51 and CSAR-HiQ_36, respectively. The summary of these datasets is displayed in Supplementary Tables S1 and S2.

The PDBbind version 2016 provides the PDB files of proteins and protein-binding pockets, as well as the SDF files of ligands. As our model only leverages 1D sequence data as inputs, we obtained the protein and pocket sequences according to their corresponding PDB files and the ligand SMILES strings by converting the SDF files. Additionally, because different samples have different sequence lengths, we needed to unify the lengths for proteins, pockets and ligands, respectively. Here, we set the lengths of proteins, pockets and SMILES as 1000, 63 and 150, respectively, and these length settings can cover around 90% data in this database. For a sequence longer than the fixed length, it was truncated, and on the contrary, it was padded with 0. The same processing procedure holds for Test2013_195, CSAR-HiQ_51 and CSAR-HiQ_36 datasets. Notably, the CSAR database does not directly provide the protein-binding pockets, so we need to analyze the PDB files of complexes to obtain the pockets. More details about the process can be found in Supplementary Text S1.

### 2.2 Input representation

**Fig. 1.**
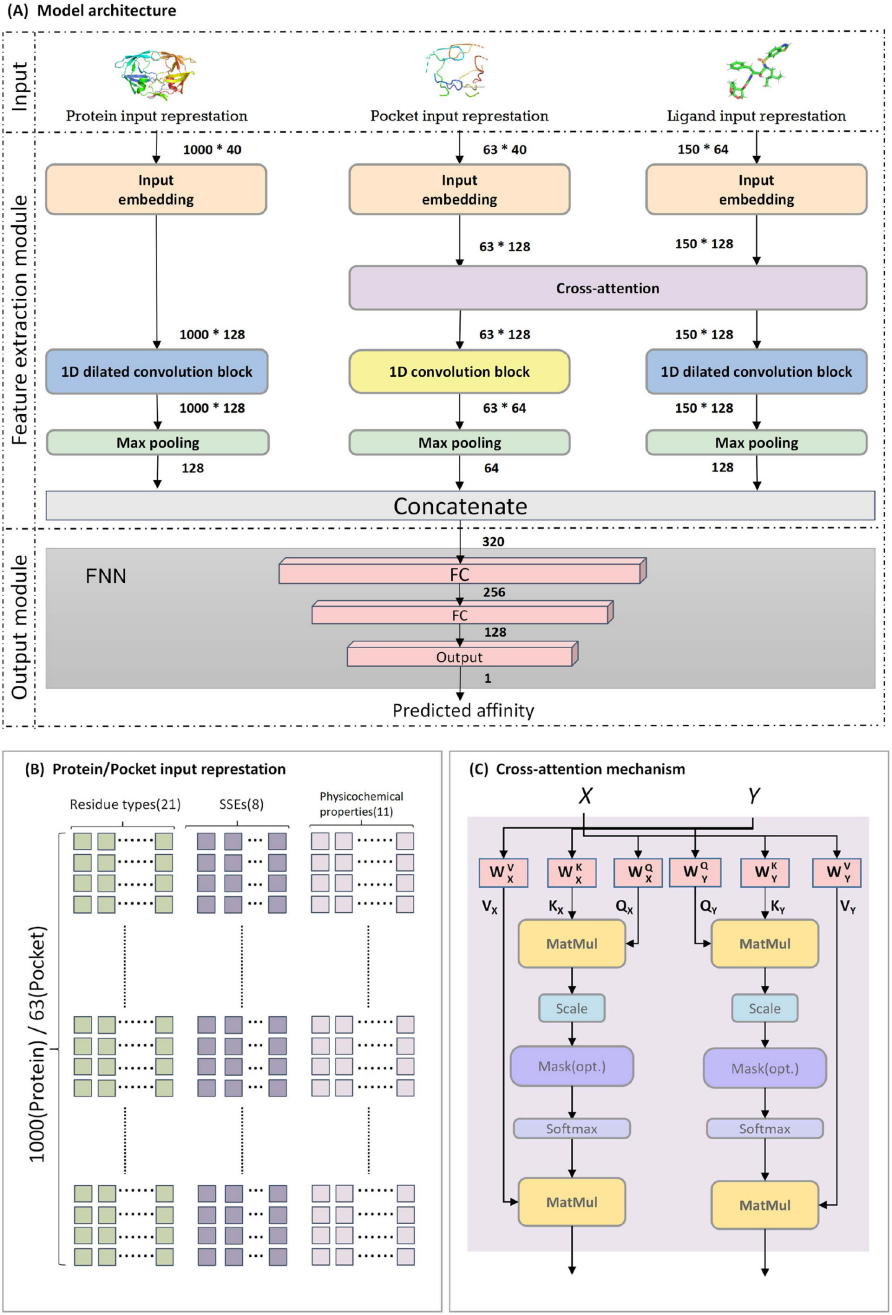
The model architecture of CAPLA. (**A**) An overall illustration of the proposed model. The number besides the arrow indicates the output dimension for each layer. (**B**) The schematic diagram of the input representations of proteins and pockets. The fixed input lengths of proteins and pockets are 1000 and 63, respectively. (**C**) The structure of a cross-attention mechanism. *X* and *Y* refer to two separate embedding sequences of each pocket and ligand, respectively

The CAPLA model contains three inputs as shown in Figure 1A, namely the input representations of proteins, pockets and ligands. In what follows, we will describe each of them in detail.

***Protein input representation*.** Figure 1B shows the input representation of a protein, consisting of the amino acid sequences, the protein SSEs and the physicochemical properties of residues. Specifically, there are generally 20 distinct kinds of amino acids and a kind of the unknown residues contained in protein sequences, so these 21 distinct kinds of residues are encoded as a 21D one-hot vector. In addition, the protein SSEs are characterized by eight states (Kabsch and Sander, 1983), i.e. $3_{10}$-helix (G), α-helix (H), Π-helix (I), β-strand (E), β-bridge (B), β-turn (T), bend (S) and coil (C), and these eight states are encoded by a 8D one-hot vector. Here, we employed the real SSEs of proteins, which were generated by the DSSP algorithm (Kabsch and Sander, 1983) from the given PDB file. The residue physicochemical properties are described by non-polar, polar, acidic, basic (Bhushan and Ali, 1987) according to their side chains, as well as seven different clusters (Shen *et al.*, 2007) based on their dipoles and side chain volumes (Supplementary Table S3). Therefore, the physicochemical properties for each residue are encoded by a 11D one-hot vector. In this way, each residue in the protein sequence is encoded by a 40D one-hot vector, and a protein sequence with a length of 1000 is encoded as a matrix of size 1000×40.

***Pocket input representation*.** The protein-binding pocket refers to the protein surface or interior cavity directly binding to the ligand, and plays important role in determining the protein–ligand binding affinity. It has been well-known that the amino acids around a pocket determine the physicochemical properties of the pocket, which along with its shape and position determines the pocket functionality (Stank *et al.*, 2016). Consequently, we leveraged the amino acid sequences of pockets as well as the SSEs and physicochemical properties as inputs. The input representation of a pocket is the same as that of a protein, yet it is worth noting that the pocket is composed of a discontinuous protein sequence containing several critical amino acids. In this work, we first employed the experimentally determined pockets as the input, and then we used the predicted pockets by P2Rank (Krivák and Hoksza, 2018) to demonstrate the general applicability of our model. In summary, a pocket sequence with a length of 63 is encoded as a matrix of size 63×40, as represented in Figure 1B.

***Ligand input representation*.** The SMILES (Weininger, 1988) on the basis atoms, bonds, etc. is usually used to represent the 1D ligand structure. Specifically, the SMILES strings of ligands are composed of 64 characters, each of which is encoded by a specific integer, e.g. ‘H’: 12, ‘C’: 42, ‘O’: 48, ‘(’: 1, ‘)’:31, ‘=’:40 and so on. Taking a SMILES string ‘C C (= O) C’ as an example, it is encoded as ‘42 42 1 40 48 31 42’. The SMILES strings of ligands were obtained by converting their corresponding SDF files through the Open Babale tool (O’Boyle *et al.*, 2011). As a result, these 64 characters are encoded as a 64D one-hot vector, and a ligand SMILES string with a length of 150 is encoded as a matrix of size 150×64.

### 2.3 The model architecture of CAPLA

The model architecture of the proposed CAPLA is depicted in Figure 1. CAPLA consists of an input layer, a feature extraction module and an output module. The input to the model has been described in the previous section, and we will describe the feature extraction module and the output module as follows.

#### 2.3.1 Feature extraction module

According to Figure 1A, the feature extraction module consists of three parts, namely protein feature extraction, pocket feature extraction and ligand feature extraction.

***Embedding layer*.** Firstly, each of the three feature extraction parts contains a linear embedding layer. The role of the embedding layer is to transform a sparse one-hot vector into a dense vector, and unify the pocket and ligand input representation to the same dimension as the inputs to the cross-attention mechanism. Here, each input representation of proteins, pockets and ligands is fed into the embedding layer to obtain a 128D dense vector. Therefore, we obtained the embedding matrices of sizes 1000×128, 63×128 and 150×128 for each protein, pocket and ligand, respectively.

***Cross-attention mechanism*.** The cross-attention mechanism was initially used in Transformer to allow each position in the decoder to cover the whole positions in the input sequence (Vaswani *et al.*, 2017). Subsequently, it was widely applied to a variety of tasks, e.g. image-text classification (Lee *et al.*, 2018) and machine translation (Gheini *et al.*, 2021). These applications have demonstrated the cross-attention mechanism enabling to construct explicit interaction between two separate inputs to fully take advantage of their correlation. Inspired by this feature, we employed the cross-attention mechanism to crossly extract features of protein-binding pockets and ligands. Moreover, based on the matrix of attention scores, the cross-attention mechanism can capture the quantifying mutual interaction between pockets and ligands, and provide support for the interpretability of the model to uncover the underlying prediction mechanism. The detailed structure of the cross-attention mechanism is illustrated in Figure 1C. Formally, the cross-attention mechanism is defined in the following way:

(1) $$\begin{array}{c} \begin{array}{cc} \mathrm{CrossAt}t_{\mathrm{pocket}} & =\mathrm{softmax}(\frac{Q_{X}K_{X}^{T}}{\sqrt{d_{k_{X}}}})V_{X}\\ & =\mathrm{softmax}(\frac{(XW_{X}^{Q}){\left(YW_{X}^{K}\right)}^{T}}{\sqrt{d_{k_{X}}}})(YW_{X}^{V}) \end{array}, \end{array}$$

(2) $$\begin{array}{c} \begin{array}{cc} \mathrm{CrossAt}t_{\mathrm{ligand}} & =\mathrm{softmax}(\frac{Q_{Y}K_{Y}^{T}}{\sqrt{d_{k_{Y}}}})V_{Y}\\ & =\mathrm{softmax}(\frac{(YW_{Y}^{Q}){\left(XW_{Y}^{K}\right)}^{T}}{\sqrt{d_{k_{Y}}}})(XW_{Y}^{V}) \end{array}, \end{array}$$

where *X* and *Y* refer to the embedding matrices of each pocket and ligand sequence, respectively. In the cross-attention mechanism for pockets, the query matrix $Q_{X}$ is calculated from *X*, and the key matrix $K_{X}$ and the value matrix $V_{X}$ are calculated from *Y*, while the cross-attention mechanism for ligands has the opposite inputs. The six matrices $W_{X}^{K}$, $W_{X}^{V}$, $W_{X}^{Q}$, $W_{Y}^{K}$, $W_{Y}^{V}$ and $W_{Y}^{Q}$ are learnable parameters, and $d_{k_{X}}$ and $d_{k_{Y}}$ denote the dimensions of matrices $K_{X}$ and $K_{Y}$, respectively. The attention score matrix $\mathrm{softmax}(\frac{Q_{X}K_{X}^{T}}{\sqrt{d_{k_{X}}}})$ or $\mathrm{softmax}(\frac{Q_{Y}K_{Y}^{T}}{\sqrt{d_{k_{Y}}}})$ indicates the similarities between each amino acid from the pocket sequence and each atom from the ligand string. In this work, we employed the multi-head cross-attention mechanism with two attention heads, so the outputs of two parallel cross-attention layers are concatenated together.

***1D dilated convolution*.** It has been demonstrated that the dilated convolution is a useful technique capable of aggregating multiscale contextual information by increasing the receptive field size of kernels with varying dilation rates (Chen *et al.*, 2018; Yu and Koltun, 2015). Motivated by this feature, we leveraged dilated convolutions to capture multiscale long-range intramolecular interactions for sequences of proteins and ligands, respectively.

In the protein feature extraction part, the dilated convolution block immediately follows the embedding layer, and contains four dilated convolution layers with the number of kernels set to 32, 64, 64 and 128, respectively. Each dilated convolution layer applies convolutions with a kernel size of 3 by setting five different dilation rates of 1, 2, 4, 8 and 16. As for the ligand feature extraction part, the dilated convolution block follows a cross-attention layer, and it consist of three dilated convolution layers with the number of kernels set to 32, 64 and 128, respectively. Similarly, each dilated convolution layer also applies convolutions with a kernel size of 3 by setting four different dilation rates of 1, 2, 4 and 8. In these two dilated convolution blocks, the number of kernels corresponding to each dilation rate in each layer is provided in Supplementary Table S4.

Additionally, because the pocket is composed of a discontinuous sequence in the protein, we leveraged 1D traditional convolutions to capture local features for pocket sequences in the pocket feature extraction part. The 1D traditional convolution block follows a cross-attention layer, and consists of three convolution layers, in which the number of kernels is set to 32, 64, 64, respectively, and the size of each kernel is set to 3.

Subsequently, each 1D dilated convolution block or 1D traditional convolution block is followed by a max pooling layer. The kernel sizes of these three max pooling layers are 1000, 63 and 150 for protein, pocket and ligand, respectively.

#### 2.3.2 Output module

The outputs of three feature extraction parts for protein, pocket and ligand are concatenated to form a vector of size 320, and then fed into an FNN to produce the final output. As shown in Figure 1, the FNN is composed of two fully connected (FC) layers and an output layer. The two FC layers contain 256 and 128 neurons, respectively, and the output layer consisting of a neuron outputs the predicted protein–ligand binding affinity. Detailed hyperparameter information of CAPLA can be found in Supplementary Table S4.

### 2.4 Model training

As the CAPLA was developed to handle the regression problem of protein–ligand binding affinity prediction, we here employed the mean squared error (MSE) loss function to train our model, which is formally defined as follows.

(3) $$\begin{array}{c} \mathrm{MSE}=\frac{1}{N}{\left(y_{i}-\hat{y_{i}}\right)}^{2}, \end{array}$$

where *N* is the total number of samples from the training data, $y_{i}$ is experimentally measured binding affinity of the sample *i* and $\hat{y_{i}}$ is the predicted binding affinity of the sample *i*. The loss function of Equation (3) is optimized by the AdamW (Gal and Ghahramani, 2016) optimization algorithm with a fixed learning rate of 0.001. After training, we selected the model with the smallest error on the validation set as the best model.

In CAPLA, the convolution and FC layers adopt PReLU (Ding *et al.*, 2018) as activation function, which helps to accelerate the training process and reduce overfitting in deep learning models (Ding *et al.*, 2018). The cross-attention mechanism layer employs GELU (Hendrycks and Gimpel, 2016) as activation function, which plays the same role as the combination of batch normalization and dropout layers. The definition of PReLU and GELU activation functions can be found in Supplementary Text S2.

The CAPLA model is implemented by CUDA 11.3 with PyTorch 1.10.2, and runs on GTX1080Ti GPU. More details about the experimental environment for the implementation of CAPLA were provided in Supplementary Figure S5.

### 2.5 Evaluation metrics

In this work, to evaluate the performance of our method and compare with other baseline methods, we adopted the widely used evaluation metrics in the protein–ligand binding affinity prediction task, including Pearson correlation coefficient (*R*), root mean square error (RMSE), mean absolute error (MAE), standard deviation (SD) and concordance index (CI). The definition of these five metrics is provided in Supplementary Text S3.

## 3 Results

In this section, we first compared our method CAPLA against state-of-the-art baseline methods for protein––ligand binding affinity prediction on various benchmarking datasets. Then, we applied CAPLA on two external independent datasets to demonstrate its generalization capability. Finally, we further provided interpretable analysis for the predicted results of CAPLA.

### 3.1 Performance of CAPLA and comparison with state-of-the-art methods

***Overall performance of CAPLA on various datasets*.** In order to evaluate the prediction performance of CAPLA, we carried out two tests on Test2016_290 and Test2013_195 sets, as listed in Table 1. As seen from Table 1, CAPLA achieves a good overall performance with *R* values higher than 0.75 and RMSE values of 1.200, 1.446 and 1.338 on the two tests and validation set, respectively. Moreover, the predicted protein–ligand binding affinities by CAPLA are highly correlated with the experimentally measured values for the two test and validation sets, with *R* values of 0.843, 0.770 and 0.771, respectively, thereby further demonstrating the preferable prediction capability of our method. The correlation between the predicted and experimentally measured binding affinities is also visualized in Supplementary Figure S1.

**Table 1.** Performance of CAPLA on different datasets

Datasets	*R* (↑)	RMSE (↓)	MAE (↓)	SD (↓)	CI (↑)
Test2016_290	0.843	1.200	0.966	1.170	0.820
Test2013_195	0.770	1.446	1.155	1.436	0.780
Validation	0.771	1.338	1.034	1.307	0.789
Training	0.867	0.968	0.755	0.931	0.840

*Note*: ↑ indicates that the greater the value, the better, and ↓ indicates that the greater the value, the worse.

Moreover, in order to investigate the contribution of different features of protein/pocket input representation in binding affinity prediction, we carried out a series of ablation experiments on Test2016_290 test set by removing the residue types, SSEs and physicochemical properties, respectively. As shown in Supplementary Table S6, the feature of residue types played a primary role in protein–ligand binding affinity prediction, while the features of SSEs and physicochemical properties can also improve the prediction capability of CAPLA for affinity prediction.

***Comparison of CAPLA with existing methods on Test2016_290 test set*.** In order to verify the effectiveness of our method in protein–ligand binding affinity prediction, we first compared the performance of CAPLA to those of eight state-of-the-art baseline methods on the Test2016_290 test set, namely DeepDTA (Öztürk *et al.*, 2018), DeepDTAF (Wang *et al.*, 2021b), Pafnucy (Stepniewska-Dziubinska *et al.*, 2018), OnionNet (Zheng *et al.*, 2019), FAST (Jones *et al.*, 2021), IGN (Jiang *et al.*, 2021), IMCP-SF (Wang and Chan, 2022) and GLI (Zhang *et al.*, 2022). Notably, among these eight compared methods, the first two are sequence-based methods, while the last six are structure-based methods. The results of comparison are displayed in Table 2, where the values of all evaluation metrics except Time were taken from their corresponding publications, while the values of Time in all models except GLI were obtained by running their source codes. Here, the metric of Time is defined as the training time of an epoch in the same dataset with a size of 11 906 samples running on our experimental environment (Supplementary Fig. S5).

**Table 2.** Comparison of the performance of CAPLA and eight baseline methods on Test2016_290 test set

Methods	*R* (↑)	RMSE (↓)	MAE (↓)	SD (↓)	CI (↑)	Time (s)(↓)
CAPLA	**0.843**	**1.200**	**0.966**	**1.170**	**0.820**	27
CAPLA-Pred	0.825	1.298	1.014	1.231	0.815	–
DeepDTA	0.749	1.443	1.148	1.445	0.771	47
DeepDTAF	0.789	1.355	1.073	1.337	0.799	**26**
Pafnucy*	0.775	1.418	1.129	1.375	0.789	2894.3
OnionNet*	0.816	1.278	–	1.450	–	587.1
FAST*	0.810	1.308	1.019	–	–	82.1
IMCP-SF*	0.791	1.452	1.155	1.349	0.790	635.9
GLI*	–	1.294	1.026	–	–	–
CAPLA (N=262)	**0.860**	**1.147**	**0.922**	**1.116**	**0.831**	–
IGN* (N=262)	0.837	1.220	0.940	1.197	0.821	99.7

*Note*: CAPLA-Pred indicates CAPLA with predicted pockets. The methods marked by * indicate structure-based ones. The best results are indicated in bold.

As observed from Table 2, CAPLA significantly outperforms the two sequence-based methods of DeepDTA and DeepDTAF in terms of all five metrics. Specifically, CAPLA yields remarkable improvement in terms of *R* and RMSE over DeepDTA by 9.4% and 16.8%, and over DeepDTAF by 5.4% and 11.4%, respectively. As our method is also sequence-based one, the comparison results demonstrate that CAPLA enables to capture more discriminative interaction features from sequence-level information of both protein and ligand alone for predicting the binding affinity.

Moreover, it can observed that CAPLA also achieves better performance over the six structure-based baseline methods including Pafnucy, OnionNet, FAST, IMCP-SF and IGN in terms of all five metrics. Specifically, CAPLA achieves improvement in terms of *R* and RMSE over Pafnucy by 6.8% and 15.4%, over OnionNet by 2.7% and 6.1%, over FAST by 3.3% and 8.3%, over IMCP-SF by 5.2% and 17.3% and over GLI by 7.3% in RMSE, respectively. In terms of the recently developed method IGN, because some complexes in Test2016_290 set cannot be represented as molecular graphs in IGN, only a subset of the Test2016_290 set consisting of 262 complexes was tested. Therefore, we also tested CAPLA on the same test subset containing 262 complexes, and directly used the test results reported in IGN. As a result, CAPLA achieves a *R* value of 0.860 and a RMSE value of 1.147 on this test subset, which is significantly better than IGN with a *R* value of 0.837 and a RMSE value of 1.220, achieving improvement of 2.3% and 6.0%, respectively. Indeed, the performance of CAPLA on the Test2016_290 test set is still better that of IGN on the subset of Test2016_290 in terms of *R* and RMSE. These results of comparison with the six structure-based methods indicate that leveraging the sequence information of proteins and ligands alone, instead of the 3D structure information of protein–ligand complexes is still sufficient to accurately estimate protein–ligand binding affinity. Moreover, it can be found from Table 2 that the sequence-based methods have more efficient computations than the structure-based methods in terms of the running time. As a result, our method CAPLA can achieve the best performance in binding affinity prediction among the compared sequence and structure-based methods.

***Comparison of CAPLA with existing methods on Test2013_195 test set*.** The Test2013_195 is also a commonly used test set to evaluate the capability of models in protein–ligand binding affinity prediction. Therefore, we also leveraged this test set to assess the prediction capability of CAPLA, and compared its prediction performance to those of DeepDTAF, Pafnucy and IGN. The comparison results are displayed in Table 3, where the values of all evaluation metrics in DeepDTAF and IGN were computed from their best models, while those in Pafnucy was directly taken from its publication (Stepniewska-Dziubinska *et al.*, 2018).

**Table 3.** Comparison of the performance of CAPLA and DeepDTAF, Pafnucy, IGN on Test2013_195 test set

Methods	*R* (↑)	RMSE (↓)	MAE (↓)	SD (↓)	CI (↑)
CAPLA	**0.770**	**1.446**	**1.154**	**1.436**	**0.780**
DeepDTAF	0.608	2.103	1.737	1.787	0.717
Pafnucy*	0.700	1.620	1.320	1.610	–
CAPLA (N=95)	**0.847**	**1.339**	1.065	**1.275**	0.816
IGN* (N=95)	0.832	1.372	**1.038**	1.328	**0.821**

*Note*: The methods marked by * indicate structure-based ones. The best results are indicated in bold.

First, it can be observed that CAPLA achieves a *R* value of 0.770 and a RMSE value of 1.446 on the Test2013_195 test set, which are substantially worse than that on the Test2016_290 test set. This may attribute to the lower-quality data of Test2013_195. However, the results of CAPLA are significantly better those of DeepDTAF, Pafnucy and IGN on Test2013_195. Specifically, with comparisons to DeepDTAF and Pafnucy in terms of *R* and RMSE, CAPLA yields remarkable improvement of 16.2% and 31.1% against DeepDTAF, along with 7% and 10.6% against Pafnucy, respectively. Similarly, IGN was tested on a subset of the Test2013_195 set consisting of 95 complexes, so we tested CAPLA on the same test subset. In comparison, CAPLA outperforms IGN on this test subset of Test2013_195 in terms of *R* and RMSE, with improvement of 1.5% and 2.4%, respectively, while performing worse than IGN in terms of MAE and CI. Consequently, these comparison results further confirm the significant prediction capability of CAPLA. Overall, the aforementioned results on Test2016_290 and Test2013_195 test sets show that CAPLA is capable of more accurately predicting protein–ligand binding affinity against other baseline methods.

***Performance of CAPLA with predicted pockets*.** The accurate protein–ligand binding pockets identified by experimental methods are of much significance in protein–ligand binding affinity prediction. However, as numerous proteins do not still have experimentally solved protein-binding pockets, in this case, we applied the predicted pockets to replace the experimental pockets as inputs to our model for predicting protein–ligand binding affinity. Here, the P2Rank (Krivák and Hoksza, 2018) tool was leveraged to predict the pockets for each protein in the training, validation and Test2016_290 test sets. First, the P2Rank predicted multiple pockets for each protein, so the pocket with the highest score was selected as the protein-binding pocket. Then, the model CAPLA was retrained on the training set and tested on the Test2016_290 test set, both of which consist of samples with predicted pockets.

The comparison results between CAPLA with predicted pockets and CAPLA with experimental pockets as well as seven baseline methods (i.e. DeepDTA, DeepDTAF, Pafnucy, OnionNet, FAST, IMCP-SF and GLI) are displayed in Table 2. Notably, the results of all six baseline methods were obtained based on the experimental pockets. As seen, the prediction performance of CAPLA becomes worse when the predicted pockets are employed as inputs, with a decrease of 1.8% and 8.2% in terms of *R* and RMSE, respectively. In spite of this, our model CAPLA with predicted pockets still outperforms the six baseline methods, and achieves improvement in terms of *R* and RMSE over DeepDTAF by 3.6% and 4.2%, over Pafnucy by 5% and 8.5%, over OnionNet by 0.9% and −1.6%, over IMCP-SF by 3.4% and 17.4% and over GLI by −0.3% in RMSE, respectively. These results demonstrate the general applicability of our method in the absence of experimental pockets.

### 3.2 Independent source affinity tests of CAPLA

In order to demonstrate the generalization capability of CAPLA, we performed two additional tests on two external datasets from independent sources (e.g. industry and academic laboratories), i.e. CSAR-HIQ_51 and CSAR-HIQ_36. We also compared the performance of CAPLA to those of DeepDTAF, Pafnucy, IGN and IMCP-SF, whose values of all evaluation metrics were computed from their best models.

As mentioned before, the CSAR database does not directly provide the protein-binding pockets, but the CAPLA model leverages the pocket as input, and hence we need to extract the residues within a certain radius of the ligand as the pocket from the PDB files of complexes in the CSAR database. In order to identify an optimal ligand radius, we established five different radiuses (i.e. 9, 10, 11, 12 and 13 Å, respectively) and evaluated their respective impacts on the prediction performance in the combination of CSAR-HIQ_51 and CSAR-HIQ_36 sets. As displayed in Supplementary Table S7, when the radius of the ligand was set to 12 Å, CAPLA achieved a better trade-off among all evaluation metrics, whereas the ligand radius was set to 13 Å, the model performance began to decrease. Consequently, we extracted the residues within 12 Å radius of the ligand as the pocket on the CSAR-HIQ datasets.

The prediction performance of CAPLA on CSAR-HIQ_51 and CSAR-HIQ_36 sets is shown in Tables 4 and 5, as well as in Supplementary Figure S2. Specifically, CAPLA achieves RMSE values of 1.848 and 1.454 on these two independent test sets, respectively. Moreover, the predicted affinities by CAPLA agree well with the experimentally measured values for CSAR-HIQ_51 and CSAR-HIQ_36 sets, with *R* values of 0.686 and 0.704, respectively. These results manifest that CAPLA is capable of generalizing well to different independent data.

**Table 4.** Comparison of the performance of CAPLA and DeepDTAF, Pafnucy, IGN, IMCP-SF on CSAR-HIQ_51 test set

Methods	*R* (↑)	RMSE (↓)	MAE (↓)	SD (↓)	CI (↑)
CAPLA	0.686	1.848	1.550	1.701	0.727
DeepDTAF	0.606	2.272	1.862	1.860	0.710
Pafnucy*	0.622	1.944	1.667	1.832	0.698
IGN*	0.417	2.263	1.714	2.125	0.657
IMCP-SF*	**0.769**	**1.629**	**1.278**	**1.491**	**0.780**

*Note*: The methods marked by * indicate structure-based ones. The best results are indicated in bold.

**Table 5.** Comparison of the performance of CAPLA and DeepDTAF, Pafnucy, IGN, IMCP-SF on CSAR-HIQ_36 test set

Methods	*R* (↑)	RMSE (↓)	MAE (↓)	SD (↓)	CI (↑)
CAPLA	**0.704**	**1.454**	**1.160**	**1.420**	**0.760**
DeepDTAF	0.543	2.765	2.318	1.679	0.670
Pafnucy*	0.566	1.658	1.291	1.649	0.566
IGN*	0.528	1.795	1.431	1.699	0.676
IMCP-SF*	0.631	1.560	1.205	1.573	0.748

*Note*: The methods marked by * indicate structure-based ones. The best results are indicated in bold.

The results of comparison of CAPLA with DeepDTAF, Pafnucy, IGN and IMCP-SF on CSAQ-HIQ_51 and CSAQ-HIQ_36 test sets are displayed in Tables 4 and 5, respectively. Specifically, on the CSAQ-HIQ_51 test set, CAPLA yields improvement in terms of *R* and RMSE over DeepDTAF by 8.0% and 18.6%, over Pafnucy by 6.4% and 4.9% and over IGN by 25.9% and 18.3%, respectively. However, our method performs worse than IMCP-SF on this test set. For the CSAQ-HIQ_36 test set, CAPLA achieves considerable improvement in terms of *R* and RMSE, i.e. over DeepDTAF by 16.1% and 47.4%, over Pafnucy by 13.8% and 12.3%, over IGN by 17.6% and 19.0% and over IMCP-SF by 7.3% and 6.7%, respectively. These comparison results indicate that the generalization capability of our method is significantly superior to those of other state-of-the-art baseline methods on different independent data.

In conclusion, the benchmark tests and the independent source tests demonstrate the superiority of CAPLA over other state-of-the-art baseline methods in protein–ligand binding affinity prediction. We consider that the superior prediction capability of CAPLA may benefit from the use of cross-attention mechanism, which can help facilitate to capture the mutual interaction features between protein-binding pockets and ligands.

### 3.3 Interpretable analysis for CAPLA

The aforementioned results demonstrated the superiority of our method CAPLA in protein–ligand binding affinity prediction. In the following, we attempted to explore the black-box mechanism of how the model makes accurate binding affinity prediction and provide an interpretable analysis for CAPLA through attention scores of protein-binding pocket sequences and ligand SMILES.

**Fig. 2.**
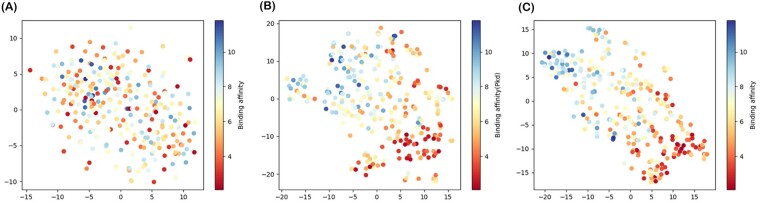
t-SNE visualization of the distribution of feature representations extracted after the embedding layer (**A**), the cross-attention layer (**B**) and the convolution layer (**C**) on Test2016_290

***Visualizing the features learned by cross-attention mechanism of CAPLA*.** In order to acquire an intuitive understanding of the features learned by CAPLA, we applied the t-Distributed Stochastic Neighbor Embedding (t-SNE) tool to map the high-dimensional feature representations (213D) extracted respectively after the embedding layer, the cross-attention layer and the convolution layer onto the 2D space as visualized in Figure 2. Specifically, Figure 2(A–C) illustrates the distribution of feature representations of all samples after the embedding layer, the cross-attention layer and the convolution layer on Test2016_290, respectively, and the distribution of these two feature representations on Test2013_195 is provided in Supplementary Figure S3. It can be observed that from Figure 2A, the samples with different binding affinities overlap and are indistinguishable in the feature space after the embedding layer, yet their embedding representations are only the learned separate characterization for pockets and ligands. While from Figure 2B, most of the samples with similar binding affinities are clustered together, along with the samples with significantly different binding affinities being separated, and more importantly, these feature representations after the cross-attention layer are the mutual interaction characterization for pockets and ligands. Moreover, based on the feature representations learned after the convolution layer of CAPLA in Figure 2C, the samples with close affinities appear to be clustered more distinctly, and the samples with different affinities are better to be separated, which suggests that the convolution layer can also further learn effective feature representations. These results confirm that the feature representation learned by the cross-attention mechanism and the convolution layer of CAPLA can effectively improve the discriminability of different protein–ligand binding affinities.

***Case studies for matching experimental results*.** To further illustrate the underlying knowledge learned by CAPLA, we visualized the attention scores of all residues in the protein-binding pocket and analyzed the key functional residues that contribute significantly to the prediction of protein–ligand binding affinity. Here, we employed the cross-attention mechanism of CAPLA to generate the attention scores for the pockets in three protein–ligand complexes (PDB IDs: 3EHY, 1O0H and 4JIA) from Test2016_260. Specifically, we first extracted the two cross-attention score matrices (150×63) generated by the two attention heads for each pocket sequence, respectively, where the query matrix *Q* refers to the embedding representation of a ligand SMILES string, and the key matrix *K* refers to the embedding representation of a pocket sequence. Then, we generated two position score vectors (1×63) for each pocket by averaging its two attention score matrices over the Query direction, respectively, and we referred to the position score vectors as attention maps. In particular, it is generally considered that in the learned attention map of a pocket, the higher the attention score of a certain residue at a specific site, the greater the contribution of the corresponding residue site to the protein–ligand binding.

**Fig. 3.**
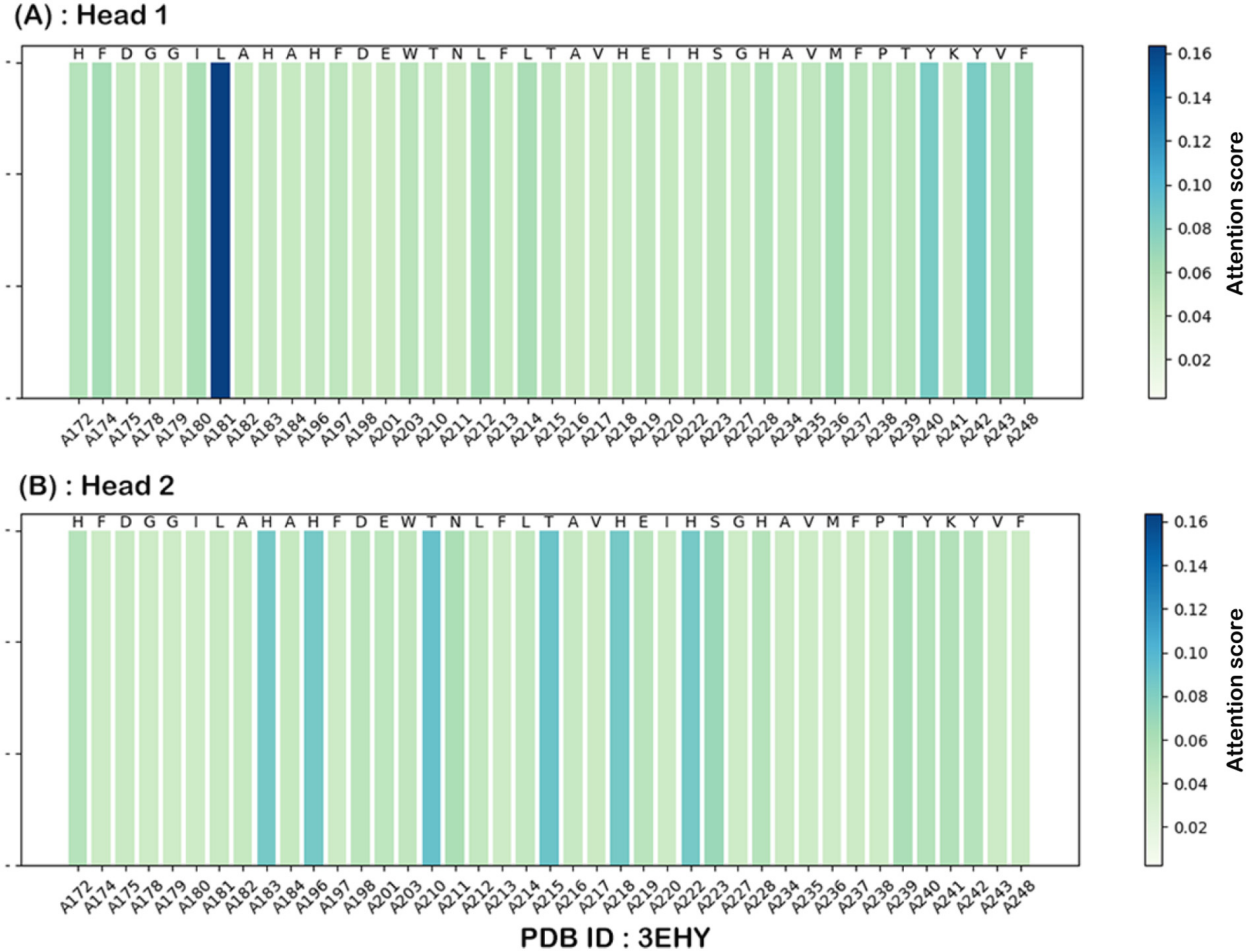
Visualization of two attention maps of the pocket in complex 3EHY learned by Head 1 (**A**) and Head 2 (**B**) in the cross-attention mechanism

**Fig. 4.**
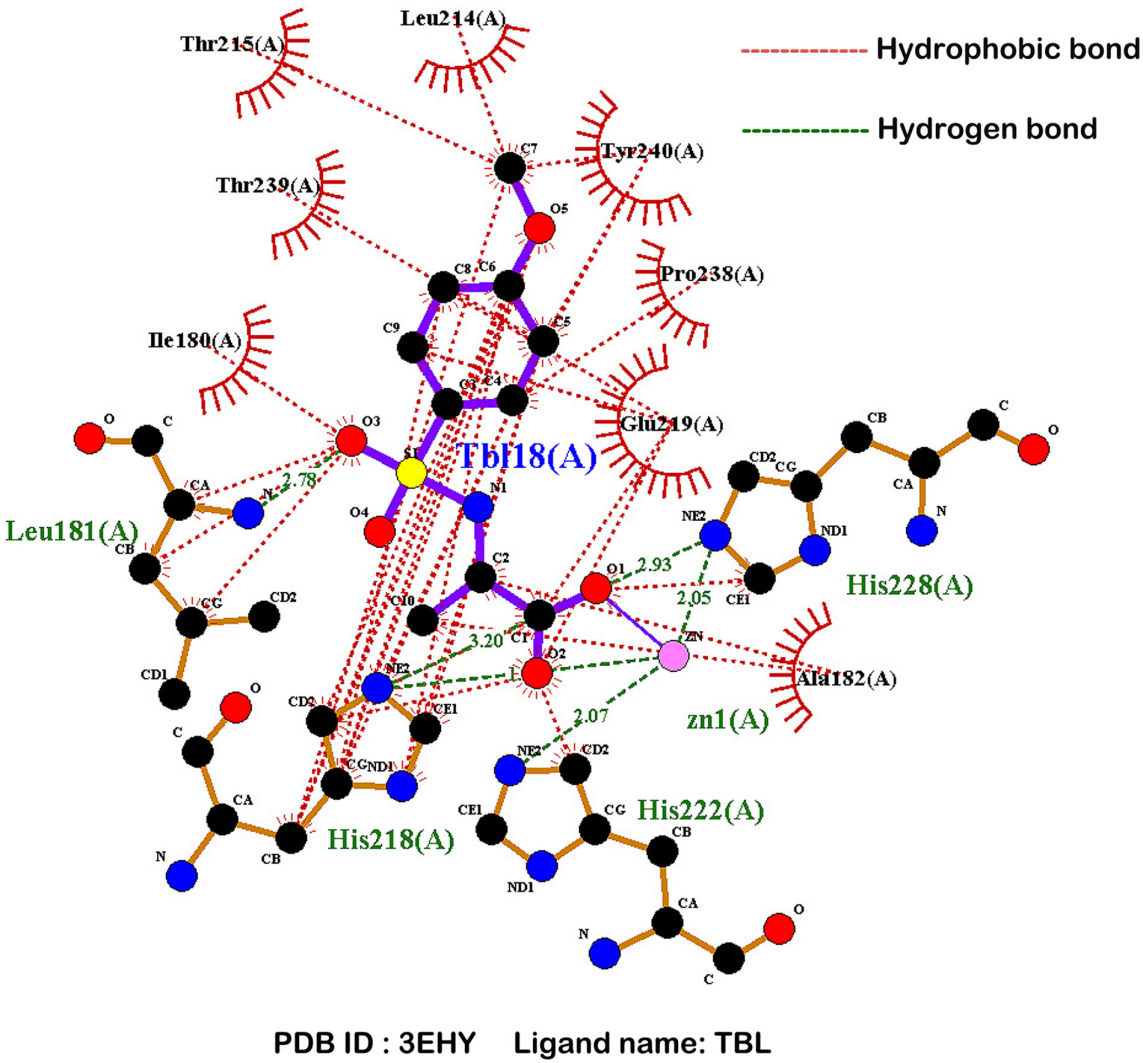
2D diagram of the hydrogen bond interactions (indicated by the dotted green lines) and the hydrophobic interactions (indicated by the dotted red lines) between pocket residues and TBL in complex 3EHY visualized by LigPlot+ program (Laskowski and Swindells, 2011)

Figure 3A and B illustrates the two attention maps of the pocket in complex 3EHY learned by Head 1 and Head 2 in the cross-attention mechanism, respectively. Indeed, the binding interaction of ligands to proteins occurs mostly through weak non-covalent interactions, such as hydrophobic interactions, hydrogen bonds, van der Waals forces (Wang *et al.*, 2002), and the former two make major contributions to the protein–ligand binding (Klebe and Böhm, 1997). As expected, our model is capable of capturing the key residues that contribute greatly to the binding through the hydrogen bond and/or hydrophobic interactions, as shown in Figure 3, including the residues Leu181, Tyr240 and Tyr242 captured by the Head 1 in the cross-attention mechanism, and the residues His183, His196, Thr210, Thr215, His218 and His222 captured by the Head 2, some of which are consistent with the experimental structure of the complex 3EHY (Dragoni *et al.*, 2009). For instance, according to the 2D diagram of pocket–ligand interaction for complex 3EHY as shown in Figure 4, the residue Leu181 of the pocket forms a hydrogen bond with the atom O3 of the ligand TBL, and it forms three hydrophobic contacts with TBL. Additionally, the residues Tyr240, Thr215, His218 and His222 of the pocket forms hydrophobic interactions with TBL, and the latter two residues also form hydrogen bond interactions with TBL. Moreover, the 3D structure (Supplementary Fig. S4) of the hydrogen bond interactions between pocket residues and TBL is further visualized by the PyMol tool (DeLano *et al.*, 2002). The other two case studies for complexes 1O0H and 4JIA can be found in Supplementary Figures S5–S10. These analysis results are supported by the experimental structural data and demonstrate that the cross-attention mechanism of CAPLA can reveal the critical residues on the pocket sequence that contribute greatly to the protein–ligand binding.

**Fig. 5.**
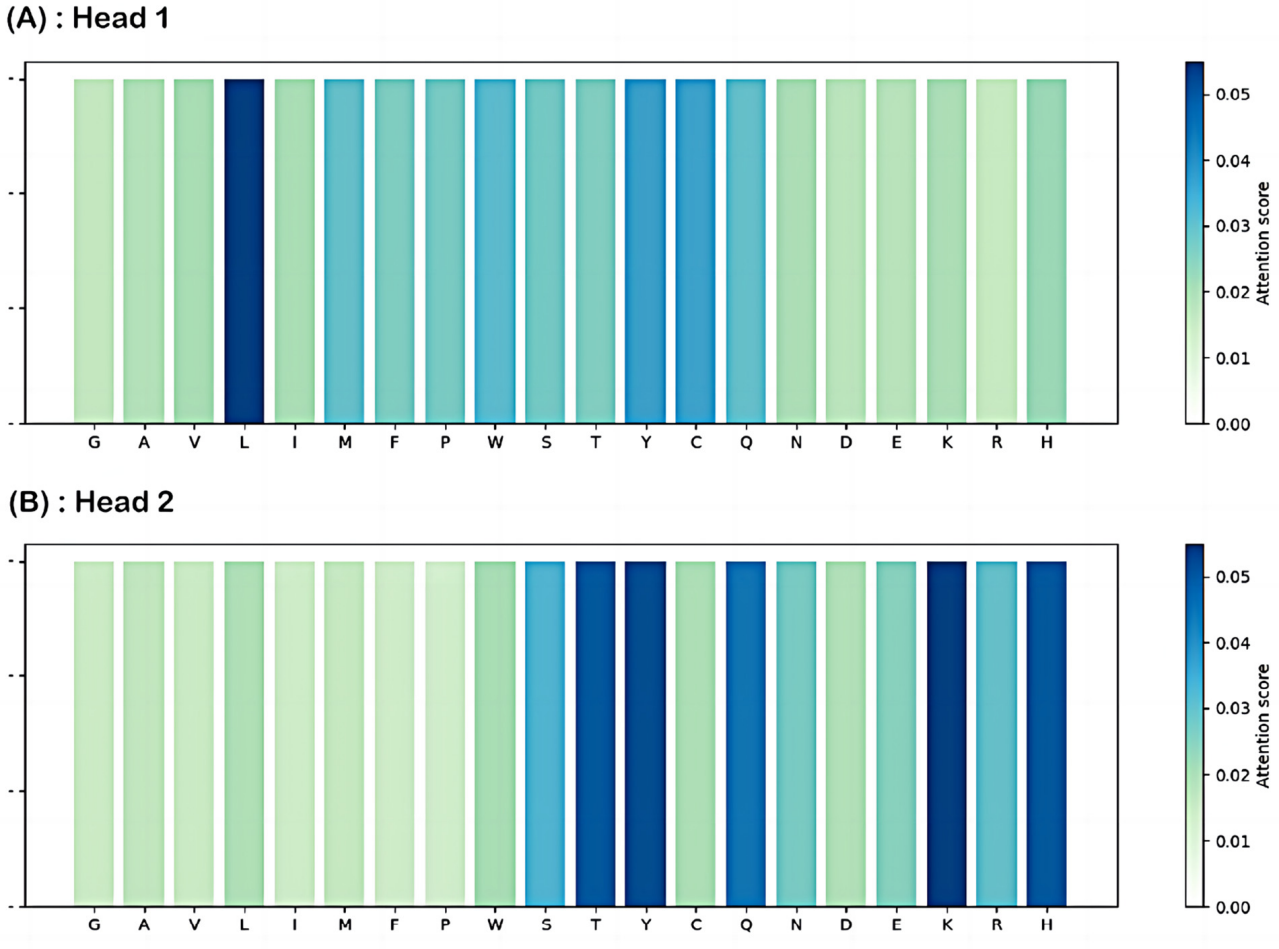
Visualization of attention scores of the amino acid types of all pocket sequences in Test2016_290 identified by Head 1 (**A**) and Head 2 (**B**) in the cross-attention mechanism

Furthermore, we explored the universal patterns detected by CAPLA in protein–ligand interaction. The attention scores of the amino acid types of all pocket sequences in Test2016_290 by Head 1 and Head 2 in the cross-attention mechanism are shown in Figure 5A and B, respectively. The attention score of each amino acid type is the average of that of the corresponding amino acid type at all pocket positions. Overall, our analyses found that CAPLA shows a preference for hydrophobic residues that usually form the binding pocket, such as the amino acids Leu, Met, Phe, Pro and Trp identified by the Head 1. Moreover, CAPLA also shows a preference for the following residues that prefer to form strong hydrogen bonds with hydroxyl groups on the ligand as reported in previous studies (Francis *et al.*, 2012; Sarkhel and Desiraju, 2004), including the amino acids Ser, Tyr, Gln, Asn, Glu, Lys, Arg and His identified by the Head 2. In addition, the residues Cys and Thr in the pockets are also capable of contributing greatly to the binding.

The findings of these functional residues may be capable of uncovering the underlying mechanism of the protein–ligand binding affinity. Furthermore, these findings also seem to be able to help better leverage CAPLA for consequent applications, e.g. drug design, etc.

## 4 Discussion and conclusion

In this work, we developed a new deep learning method based on the cross-attention mechanism, called CAPLA, for improved prediction of protein–ligand binding affinity using sequence information of both protein and ligand alone. In particular, CAPLA leverages the cross-attention mechanism to capture the intermolecular correlation of protein-binding pockets and ligands. Comprehensive experiments on various benchmarking datasets demonstrated the superior prediction capability of CAPLA over other state-of-the-art baseline methods. In addition, we demonstrated that CAPLA is capable of revealing the critical residues on the pocket sequence that make greater contributions to the protein–ligand binding through the cross-attention mechanism.

There is still some future work that might further improve our method, summarized as follows. First, CAPLA leverages sequence information of proteins and pockets only as the model inputs. Therefore, it is interesting and promising to introduce the 3D structure information of protein–ligand complexes in the model. Second, the input representation of a ligand consists of its SMILES string alone. In the future, we can incorporate more feature representation of ligands into our model, e.g. ligand structures and atom physicochemical properties. Moreover, it is worthwhile to explore the application potentials of CAPLA in drug discovery.

## Supplementary Material

btad049_Supplementary_Data
