# Supplementary material for: CAPLA: improved prediction of protein–ligand binding affinity by a deep learning approach based on a cross-attention mechanism
Source: Bioinformatics. 2023 Jan 23;39(2):btad049. doi: 10.1093/bioinformatics/btad049 (PMC9900214; doi:10.1093/bioinformatics/btad049)
Supplement: btad049_Supplementary_Data [file btad049_supplementary_data.zip › Supplementary Material_CAPLA_revised.docx]

Supplemental Information

**Table S1.** PDB ID of protein-ligand complexes in each dataset.

| **Datasets** | **PDB ID** |
| --- | --- |
| **Test2016_290** | 4dld,1a30,3lka,4dli,4e5w,4e6q,4ea2,2zb1,2zcq,2zcr,2zda,1sqa,1nc1,1nc3,4pcs,3syr,3fur,3fv1,3fv2,3g0w,4wiv,2r9w,4agn,4agp,4agq,3ozs,3ozt,3p5o,3dd0,2br1,2brb,1qkt,1r5y,2wer,2wn9,2wnc,2wtv,2wvt,5a7b,5aba,2p15,2p4y,4mme,4mrw,4mrz,4msc,4msn,4rfm,3utu,3uuo,1bzc,1owh,1oyt,1p1n,1p1q,1ps3,1pxn,1u1b,1uto,1k1i,3r88,3rlr,3bgz,3n86,3k5v,3kgp,3kr8,3kwa,2fvd,2fxs,4f9w,3uri,3ehy,3ejr,2vkm,1z6e,1z95,1z9g,5dwr,3gv9,3gy4,4ddh,4ddk,2xnb,2xys,2y5h,4llx,3tsk,3twp,3u5j,3u8k,3u8n,3wz8,3zdg,3zso,3zsx,3zt2,4j3l,4jfs,4jia,4jsz,4tmn,4twp,4ty7,4u4s,1o3f,1o5b,4qac,4qd6,3cj4,3coy,3coz,3cyx,1lpg,1bcu,1syi,3ag9,3ao4,3arp,3arq,3aru,3arv,3prs,3pww,3pxf,3pyy,3l7b,3mss,3myg,3n76,3n7a,1vso,1w4o,2qnq,3o9i,3oe4,3oe5,2vvn,3b68,1gpk,1gpn,1h22,1h23,3wtj,2xbv,2xdl,2xii,2xj7,1q8t,1q8u,1qf1,4gr0,4hge,2v00,2v7a,4ih5,4ih7,4ivb,4ivc,4ivd,4j21,2vw5,2w4x,2w66,2wbg,2wca,2weg,4eky,4eo8,4eor,4w9c,4w9h,4w9i,4gkm,4j28,4w9l,3ivg,3jvr,3jvs,3jya,3g2n,3g2z,3g31,3gbb,3gc5,3ge7,3gnw,3gr2,3f3a,3f3c,3f3d,3f3e,3fcq,1nvq,1o0h,2al5,4lzs,4m0y,4m0z,4mgd,1y6r,1yc1,1ydr,1ydt,4abg,2yfe,2yge,2yki,2ymd,3u9q,3udh,3ueu,3uev,3uew,3uex,3ui7,3uo4,3up2,3d4z,3d6q,4ogj,4owm,4jxs,4k18,4k77,4kz6,4kzq,4kzu,4de1,4de2,4de3,4djv,1c5z,5c1w,5c28,5c2h,4bkt,3dx1,3dx2,3dxg,3e5a,3ebp,4gfm,4gid,4cig,4ciw,4cr9,4cra,4crc,2iwx,2j78,2j7h,1mq6,5tmn,1eby,4x6p,2x00,2xb8,2zy1,3acw,2hb1,3bv9,2c3i,2cbv,2cet,2pog,1s38,3rr4,3rsx,3ryj,4f09,4f2w,4f3c,3qgy,3qqs,3ary,3b1m,3b27,3b5r,3b65,3nq9,3nw9,3nx7,2qbp,2qbq,2qbr,2qe4,1e66,3e92,3e93 |
| **Test2013_195** | 10gs,1a30,1bcu,1e66,1f8b,1f8c,1f8d,1gpk,1h23,1hfs,1hnn,1igj,1jyq,1kel,1lbk,1lol,1loq,1lor,1mq6,1n1m,1n2v,1nvq,1o3f,1o5b,1os0,1oyt,1p1q,1ps3,1q8t,1q8u,1qi0,1r5y,1sln,1sqa,1u1b,1u33,1uto,1vso,1w3k,1w3l,1w4o,1xd0,1yc1,1z95,1zea,2brb,2cbj,2cet,2d1o,2d3u,2fvd,2g70,2gss,2hb1,2iwx,2j62,2j78,2jdm,2jdu,2jdy,2obf,2ole,2p4y,2pcp,2pq9,2qbp,2qbr,2qft,2qmj,2r23,2v00,2v7a,2vl4,2vo5,2vot,2vvn,2vw5,2w66,2wbg,2wca,2weg,2wtv,2x00,2x0y,2x8z,2x97,2xb8,2xbv,2xdl,2xhm,2xnb,2xy9,2xys,2y5h,2yfe,2yge,2yki,2ymd,2zcq,2zcr,2zjw,2zwz,2zx6,2zxd,3acw,3ag9,3ao4,3b3s,3b3w,3b68,3bfu,3bkk,3bpc,3cft,3cj2,3coy,3cyx,3d4z,3dd0,3dxg,3e93,3ebp,3ehy,3ejr,3f17,3f3a,3f3c,3f3e,3f80,3fcq,3fk1,3fv1,3g0w,3g2n,3g2z,3gbb,3gcs,3ge7,3gnw,3gy4,3huc,3i3b,3imc,3ivg,3jvs,3k5v,3kgp,3kv2,3kwa,3l3n,3l4u,3l4w,3l7b,3lka,3mfv,3mss,3muz,3myg,3n7a,3n86,3nox,3nq3,3nw9,3oe5,3ov1,3owj,3ozt,3pe2,3pww,3pxf,3s8o,3su2,3su3,3su5,3u9q,3udh,3ueu,3uex,3uo4,3uri,3utu,3vd4,3vh9,3zso,3zsx,4de1,4de2,4des,4dew,4djr,4djv,4g8m,4gid,4gqq,4tmn |
| **Test2016_262** | 1bcu,1bzc,1c5z,1e66,1eby,1gpn,1h22,1h23,1k1i,1lpg,1mq6,1nc1,1nc3,1nvq,1o0h,1o3f,1o5b,1owh,1oyt,1pxn,1q8t,1q8u,1qf1,1qkt,1r5y,1s38,1sqa,1syi,1u1b,1uto,1vso,1w4o,1y6r,1yc1,1ydr,1ydt,1z6e,1z95,1z9g,2al5,2br1,2brb,2c3i,2cbv,2cet,2fvd,2fxs,2hb1,2iwx,2j78,2j7h,2p15,2p4y,2pog,2qbp,2qbq,2qbr,2qe4,2qnq,2v00,2v7a,2vkm,2vvn,2vw5,2w4x,2w66,2wbg,2wca,2weg,2wn9,2wnc,2wtv,2wvt,2x00,2xb8,2xbv,2xdl,2xii,2xj7,2xnb,2xys,2y5h,2yfe,2yge,2yki,2zb1,2zcq,2zcr,2zda,2zy1,3acw,3ao4,3arp,3arq,3aru,3arv,3ary,3b1m,3b27,3b5r,3b65,3b68,3bgz,3cj4,3coy,3coz,3cyx,3d4z,3d6q,3dd0,3dx2,3dxg,3e5a,3e92,3e93,3ebp,3ehy,3ejr,3fcq,3fur,3g0w,3g2n,3g2z,3g31,3gc5,3gnw,3gv9,3gy4,3jvr,3jvs,3jya,3k5v,3kgp,3kr8,3kwa,3l7b,3lka,3mss,3myg,3n76,3n7a,3n86,3nq9,3nw9,3nx7,3o9i,3oe4,3oe5,3ozs,3ozt,3p5o,3prs,3pww,3pxf,3pyy,3qgy,3qqs,3r88,3rlr,3rr4,3rsx,3ryj,3syr,3tsk,3twp,3u5j,3u8k,3u8n,3u9q,3udh,3ueu,3uex,3ui7,3uo4,3up2,3utu,3uuo,3wtj,3wz8,3zdg,3zso,3zsx,3zt2,4abg,4agn,4agp,4agq,4bkt,4cig,4ciw,4cr9,4cra,4crc,4ddh,4ddk,4de1,4de2,4de3,4djv,4dld,4dli,4e5w,4e6q,4ea2,4eky,4eo8,4eor,4f09,4f2w,4f3c,4f9w,4gfm,4gid,4gkm,4gr0,4hge,4ih5,4ih7,4ivb,4ivc,4ivd,4j21,4j28,4j3l,4jfs,4jia,4jsz,4jxs,4k77,4kz6,4kzq,4kzu,4llx,4lzs,4m0y,4m0z,4mgd,4mme,4mrw,4mrz,4msc,4msn,4ogj,4owm,4pcs,4qac,4qd6,4rfm,4twp,4ty7,4u4s,4w9c,4w9h,4w9l,4wiv,4x6p,5a7b,5aba,5c1w,5c28,5c2h,5dwr |
| **Test2013_95** | 1bcu,1e66,1h23,1mq6,1nvq,1o3f,1o5b,1oyt,1q8t,1q8u,1r5y,1sqa,1u1b,1uto,1vso,1w4o,1yc1,1z95,2brb,2cet,2fvd,2hb1,2iwx,2j78,2p4y,2qbp,2qbr,2v00,2v7a,2vvn,2vw5,2w66,2wbg,2wca,2weg,2wtv,2x00,2xb8,2xbv,2xdl,2xys,2y5h,2yfe,2yge,2yki,2zcq,2zcr,3acw,3ao4,3b68,3coy,3cyx,3d4z,3dd0,3dxg,3e93,3ebp,3ehy,3ejr,3fcq,3g0w,3g2n,3g2z,3gnw,3gy4,3jvs,3k5v,3kgp,3l7b,3lka,3mss,3myg,3n7a,3n86,3nw9,3oe5,3ozt,3pxf,3u9q,3udh,3ueu,3uex,3uo4,3utu,3zso,3zsx,4de1,4de2,4djv,4gid,1ps3,3ge7,3i3b,3l3n,3muz |
| **CSAR-HIQ_51** | 1w6o,2arb,2are,2pog,1vso,2b3f,2bbf,2idz,2ihk,2ilz,2jff,2otz,2ou0,2pjo,2pzv,2q3c,2qeh,2qvu,2r3d,2rde,2v8q,3bgz,2v8y,2jgb,1uto,1ukb,2cjp,2add,2jdu,2jdy,2p3t,2p4y,2q6m,2qbq,2qbr,2qmj,2qnq,2rca,2v7t,2v7u,2v7v,2vhw,2vkm,2vw5,2z8f,2zlz,3c7i,3e92,3ene,3f3d,3f4j |
| **CSAR-HIQ_36** | 1gpk,1h22,1h23,1hnn,1nc1,1nc3,1p1n,1ps3,1r5y,1s38,1syi,10gs,1yc1,1z95,2brb,2d3u,2fvd,2hb1,2iwx,2j78,1eby,1qkt,1w3k,1xl5,1y93,4ubp,1q0y,1s7y,1txf,1uld,1uzv,1zhx,2dm5,1q6e,1q6g,1xw6 |

**Table S2.** Summary of datasets.

| **Dataset** | **Data source** | **Number of complexes** |
| --- | --- | --- |
| **Training** | PDBbind version 2016 | 11906 |
| **Validation** | PDBbind version 2016 | 1000 |
| **Test2016_290** | PDBbind version 2016 | 290 |
| **Test2013_195** | PDBbind version 2013 | 195 |
| **CSAR-HiQ_51** | CSAR NRC-HIQ | 51 |
| **CSAR-HiQ_36** | CSAR NRC-HIQ | 36 |

**Table S3.** Seven different clusters based on their dipoles and side chain volumes.

| **Classification** | **Dipole scale^a^** | **Volume scale^b^** | **Amino acids** |
| --- | --- | --- | --- |
| **1** | +^c^ | + | Cys |
| **2** | +’+’+’ | + | Asp, Glu |
| **3** | +++ | + | Arg, Lys |
| **4** | ++ | + | His, Asn, Gln, Trp |
| **5** | + | + | Tyr, Met, Thr, Ser |
| **6** | - | + | Ile, Leu, Phe, Pro |
| **7** | - | - | Ala, Gly, Val |

^a^ Dipole scale (Debye): -, Dipole<1.0; +, 1.0<Dipole<2.0; ++, 2.0<Dipole<3.0; +++, Dipole>3.0; +’+’+’, Dipole>3.0 with opposite orientation.

^b^ Volume scale (Å^3^): -, Volume<50; +, Volume>50.

^c^ Cys is separated from classification 5 because of its ability of form disulfide bonds.

**Table S4.** The hyperparameter information of CAPLA.

| **Hyperparameter** | **Value** | |
| --- | --- | --- |
| **The dimension of linear embedding of protein and pocket** | 128 | |
| **The dimension of linear embedding of ligand** | 128 | |
| **The number of neurons in the full connected layer of the cross-attention mechanism** | 128 | |
| **Dropout rate in the full connected layer of the cross-attention mechanism** | 0.1 | |
| **The number of heads in the cross-attention mechanism** | 2 | |
| **Dropout rate after Q×V operation in the cross-attention mechanism** | 0.1 | |
| **The number of kernels for** **dilated convolution block in protein feature extraction part.** **Dilation rates of [1, 2, 4, 8, 16]** | **Layer 1** | 8, 6, 6, 6, 6 (sum = 32) |
|  | **Layer 2** | 16, 12, 12, 12, 12 (sum = 64) |
|  | **Layer 3** | 16, 12, 12, 12, 12 (sum = 64) |
|  | **Layer 4** | 32, 24, 24, 24, 24 (sum = 128) |
| **The number of kernels for dilated convolution block in ligand feature extraction part.** **Dilation rates of [1, 2, 4, 8]** | **Layer 1** | 8, 8, 8, 8 (sum = 32) |
|  | **Layer 2** | 16, 16, 16, 16 (sum = 64) |
|  | **Layer 3** | 32, 32, 32, 32 (sum = 128) |
| **The number of kernels for traditional convolution block in pocket feature extraction part** | **Layer 1** | 32 |
|  | **Layer 2** | 32 |
|  | **Layer 3** | 64 |
| **Dropout rate after concatenate operation** | 0.2 | |
| **The number of neurons in each full connected layer of FNN** | 256, 128, 1 | |
| **Dropout rate in each full connected layer of FNN** | 0.5 | |

**Table S5.** The experimental environment for the implementation of CAPLA.

| **Hardware and software** | **Experimental environment** |
| --- | --- |
| GPU | GEFORCE GTX1080Ti (RAM:11GB, 11GHZ) |
| CPU | Intel(R) Xeon(R) CPU E5-2620 v4 (2.10GHz, 16 Cores) |
| RAM | 128 GB |
| Python version | Python 3.6.5 |
| PyTorch version | Torch 1.10.2 |
| CUDA version | CUDA 11.3 |

**Table S6.** The impacts of different features of protein/pocket input representation on the prediction performance of CAPLA on Test2016_290 test set.

| **Features** | **R↑** | **RMSE↓** | **MAE↓** | **SD↓** | **CI↑** |
| --- | --- | --- | --- | --- | --- |
| **Without residue types** | 0.784 | 1.553 | 1.241 | 1.350 | 0.790 |
| **Without SSEs** | 0.819 | 1.256 | 0.994 | 1.248 | 0.811 |
| **Without physicochemical properties** | 0.808 | 1.304 | 1.059 | 1.282 | 0.802 |
| **CAPLA** | **0.843** | **1.200** | **0.966** | **1.170** | **0.820** |

Note: The best results are indicated in bold.

**Table S7.** The impacts of different radiuses of the ligand as the pocket on the prediction performance of CAPLA in the combination of CSAR-HIQ_51 and CSAR-HIQ_36 sets.

| **Radius of the ligand** | **R↑** | **RMSE↓** | **MAE↓** | **SD↓** | **CI↑** |
| --- | --- | --- | --- | --- | --- |
| **9Å** | 0.679 | **1.667** | **1.378** | 1.618 | 0.726 |
| **10Å** | 0.663 | 1.725 | 1.416 | 1.650 | 0.722 |
| **11Å** | 0.673 | 1.716 | 1.404 | 1.630 | 0.726 |
| **12Å** | **0.691** | 1.696 | 1.389 | **1.594** | **0.735** |
| **13Å** | 0.690 | 1.718 | 1.383 | 1.595 | 0.733 |

Note: The best results are indicated in bold.

**Text S1.** The details of the process of obtaining the pockets for CSAR-HIQ dataset.

The CSAR database does not directly provide the protein-binding pockets, so we need to analyze the PDB files of complexes to obtain the pockets. Specifically, we used the Chimera tool to select the residues within a X (9, 10, 11, 12, 13)-Å radius of the ligand as the pocket, as shown in the following figure.


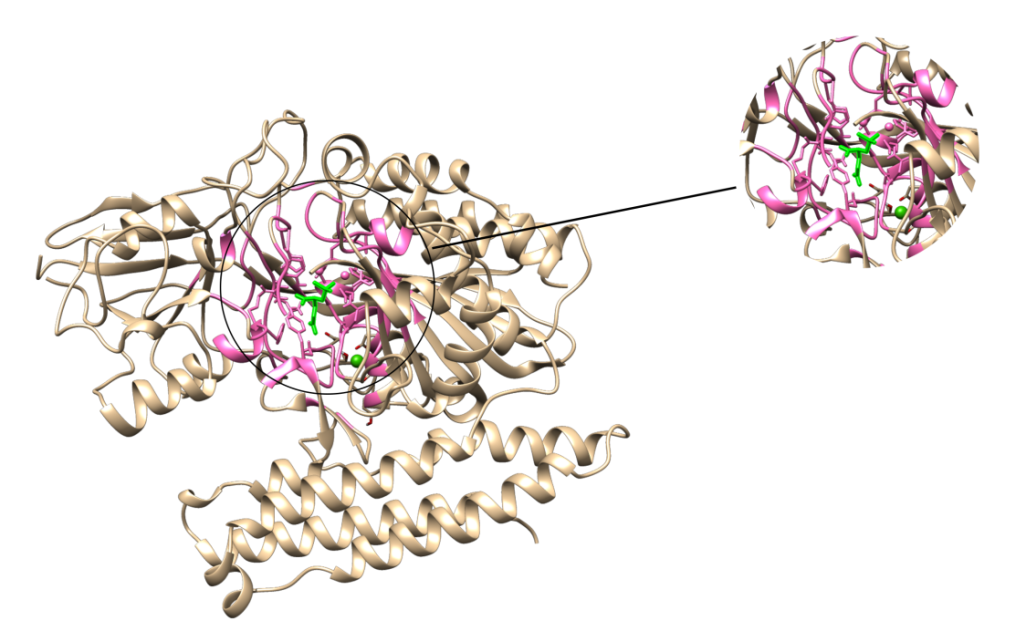


**Text S2.** The definition of PReLU and GELU activation functions.

The **PReLU** (Parametric Rectified Linear Unit) activation function is defined as follows:

$$\mathrm{PReLU}\left( x \right)= \left\{ \begin{aligned} 0 x\leq0 \\ ax x>0 \end{aligned} \right.$$

where *a* is a learnable parameter. This learned parameter can accelerate the learning speed of the model and reducing overfitting.

The **GELU** (GAUSSIAN ERROR LINEAR UNIT) activation function is defined as follows:

$$\mathrm{GELU}\left（ X \right）=xP\left( X\leq x \right)=x\Phi\left( x \right)$$

$$\approx0.5x(1+\tanh\left[ \sqrt{\frac{2}{\pi}}(x+0.044715x^{3}) \right])$$

where $\Phi(x)$ is a cumulative distribution of Gaussian distribution.

**Text S3.** The definition of the five evaluation metrics used in this work.

The Pearson correlation coefficient (**R**), root mean square error (**RMSE**), and mean absolute error (**MAE**) are defined as follows:

$$R=\frac{\sum_{i=1}^{n} \left( {true}_{i}-\bar{true} \right)\left( {predict}_{i}-\bar{predict} \right)}{\sqrt{\sum_{i=1}^{n} \left( {true}_{i}-\bar{true} \right)}\sqrt{\sum_{i=1}^{n} \left( {predict}_{i}-\bar{predict} \right)}}$$

$$RMSE=\sqrt{\frac{1}{n}\sum_{i=1}^{n} \left( {true}_{i}-{predict}_{i} \right)^{2}}$$

$$MAE= \frac{1}{n}\sum_{i=1}^{n} \left| {true}_{i}- {predict}_{i} \right|$$

where *n* is the number of protein–ligand complexes, ${true}_{i}$ refers to the experimentally measured binding affinity of the sample indexed with *i*, and ${predict}_{i}$ refers to the predicted binding affinity of the sample indexed with *i*, $\bar{true}$ refers to the mean of the experimentally measured binding affinity of n samples, and $\bar{predict}$ refers to the mean of the predicted binding affinity of *n* samples.

The standard deviation (**SD**) is defined as follows:

$$SD=\sqrt{\frac{1}{n-1}\sum_{i=1}^{n} \left[ {true}_{\dot{i}}-\left( a{*predict}_{i}+b \right) \right]^{2}}$$

where *n* is the number of protein-ligand complexes, ${true}_{i}$ and ${predict}_{i}$ are the actual and predicted binding affinities of the sample *i*, respectively. *a* and *b* are slope and intercept of the function line between actual and predicted values.

The concordance index (**CI**) is defined as follows:

$CI= \frac{1}{Z}\sum_{{true}_{i}>{true}_{j}} h\left( {predict}_{i}-{predict}_{j} \right)$

$$h\left( x \right) = \left\{ \begin{aligned} 1 x>0 \\ 0.5 x=0 \\ 0 x<0 \end{aligned} \right.$$

where ${predict}_{i}$ is the predicted value for the larger actual binding affinity value ${true}_{i}$ and ${predict}_{j}$ is the predicted value for the smaller actual affinity value ${true}_{j}$. The normalization constant Z is the total number of protein-ligand complexes.


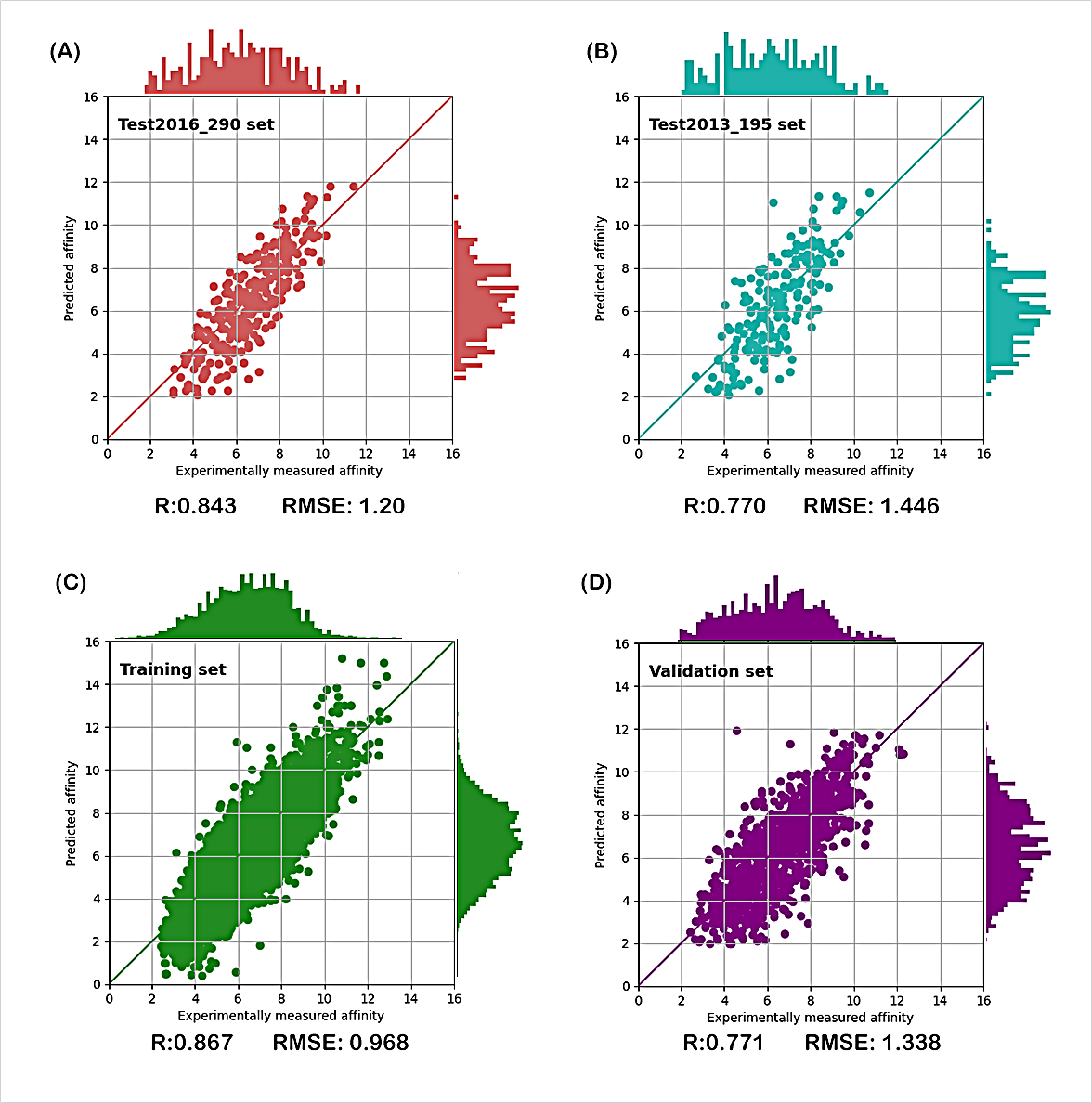


**Figure S1.** Scatter plots of the predicted affinities by CAPLA against the experimentally measured affinities for Test2016_290 test set (A), Test2013_195 test set (B), training set (C), and validation set (D).

**
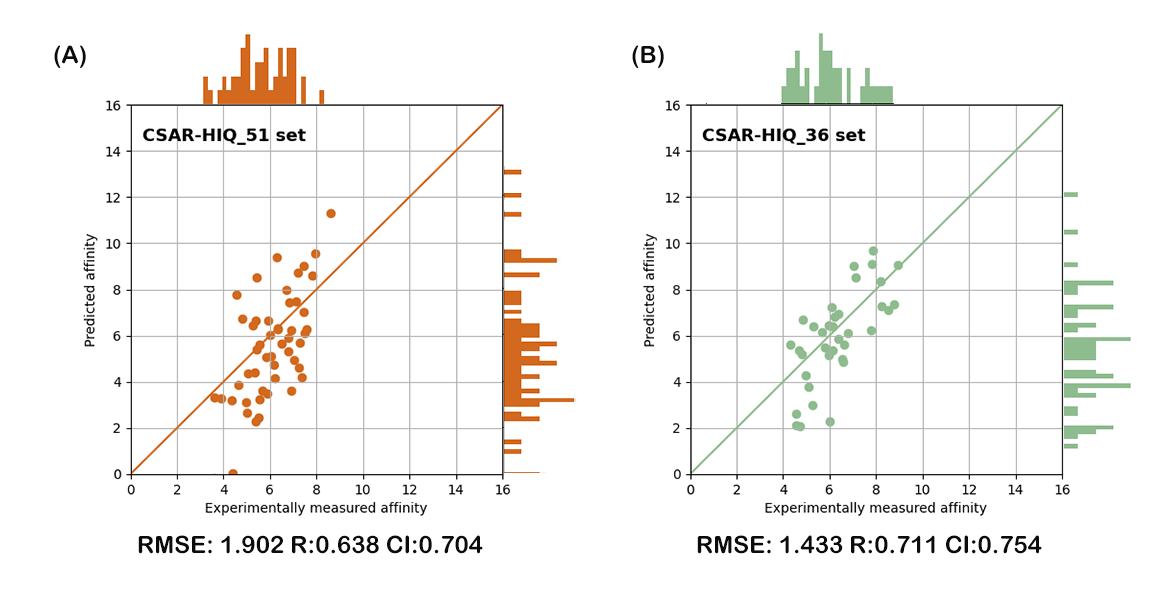
**

**Figure S2.** Scatter plots of the predicted affinities by CAPLA against the experimentally measured affinities for CSAQ-HIQ_51 set (A), and CSAQ-HIQ_36 set (B).


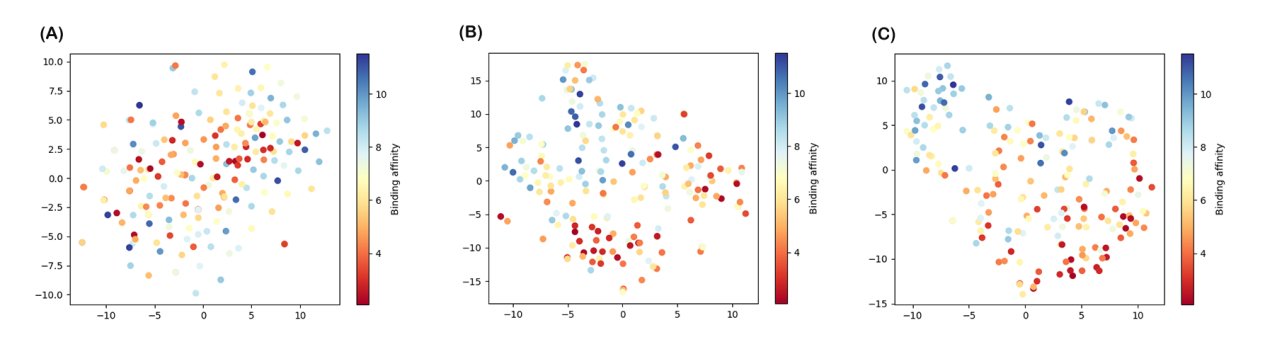


**Figure S3.** t-SNE visualization of the distribution of feature representations extracted after the embedding layer (A), the cross-attention layer (B), and the convolution layer (C) on Test2013_195.


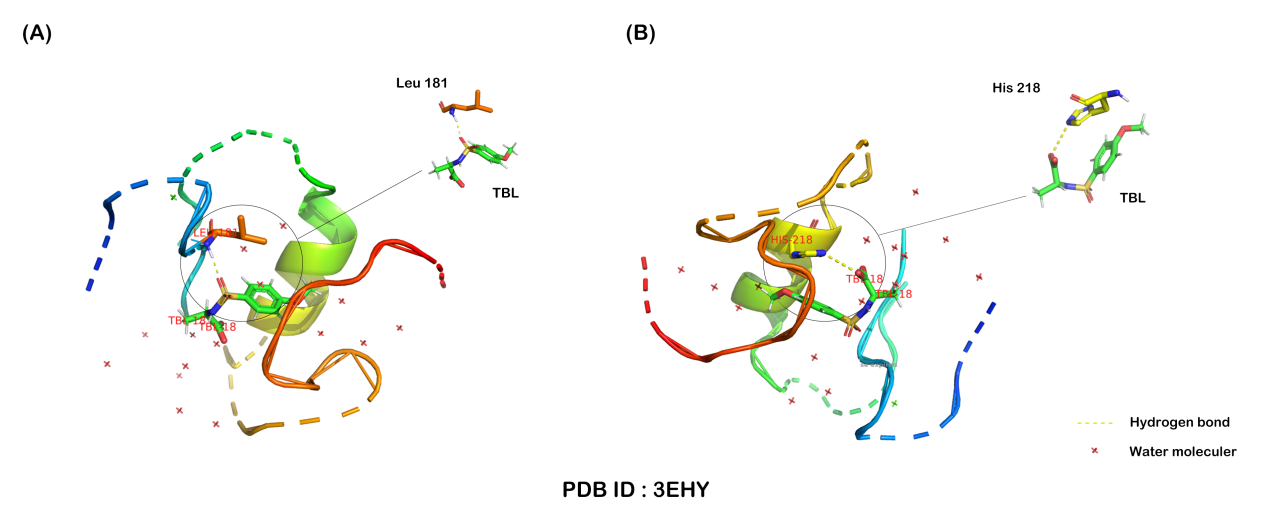


**Figure S4.** The 3D structure of the hydrogen interactions between pocket residues Leu181 (A), His218 (B) and the ligand TBL in complex 3EHY visualized by PyMol tool.


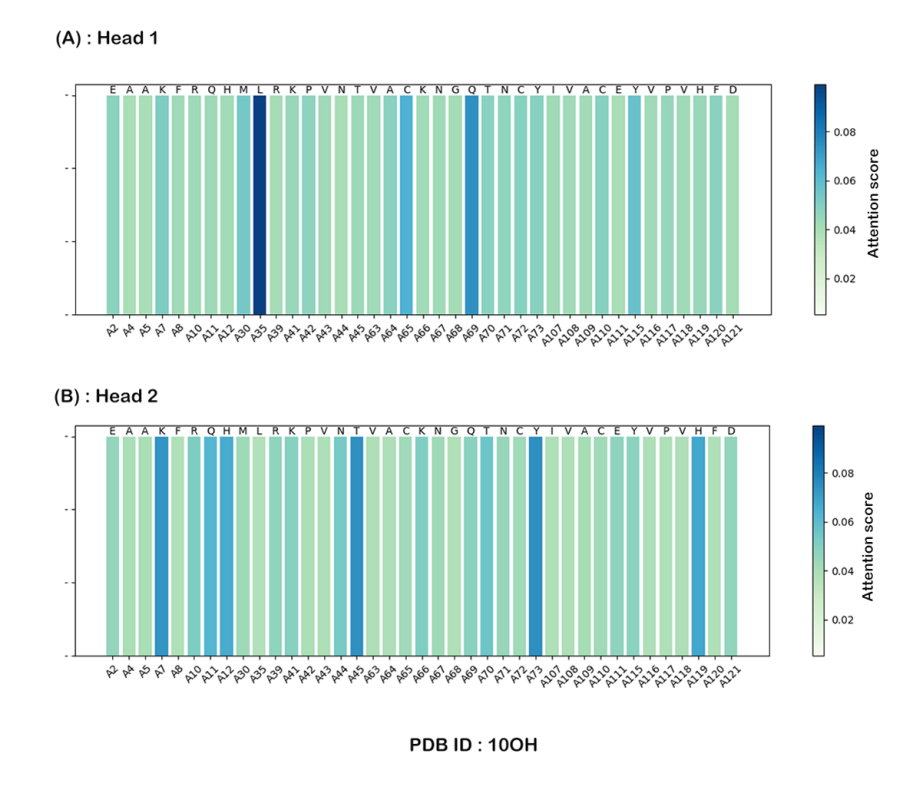


**Figure S5.** Visualization of two attention maps of the pocket in complex 10OH learned by head 1 (A) and head 2 (B) in the cross-attention mechanism.

**
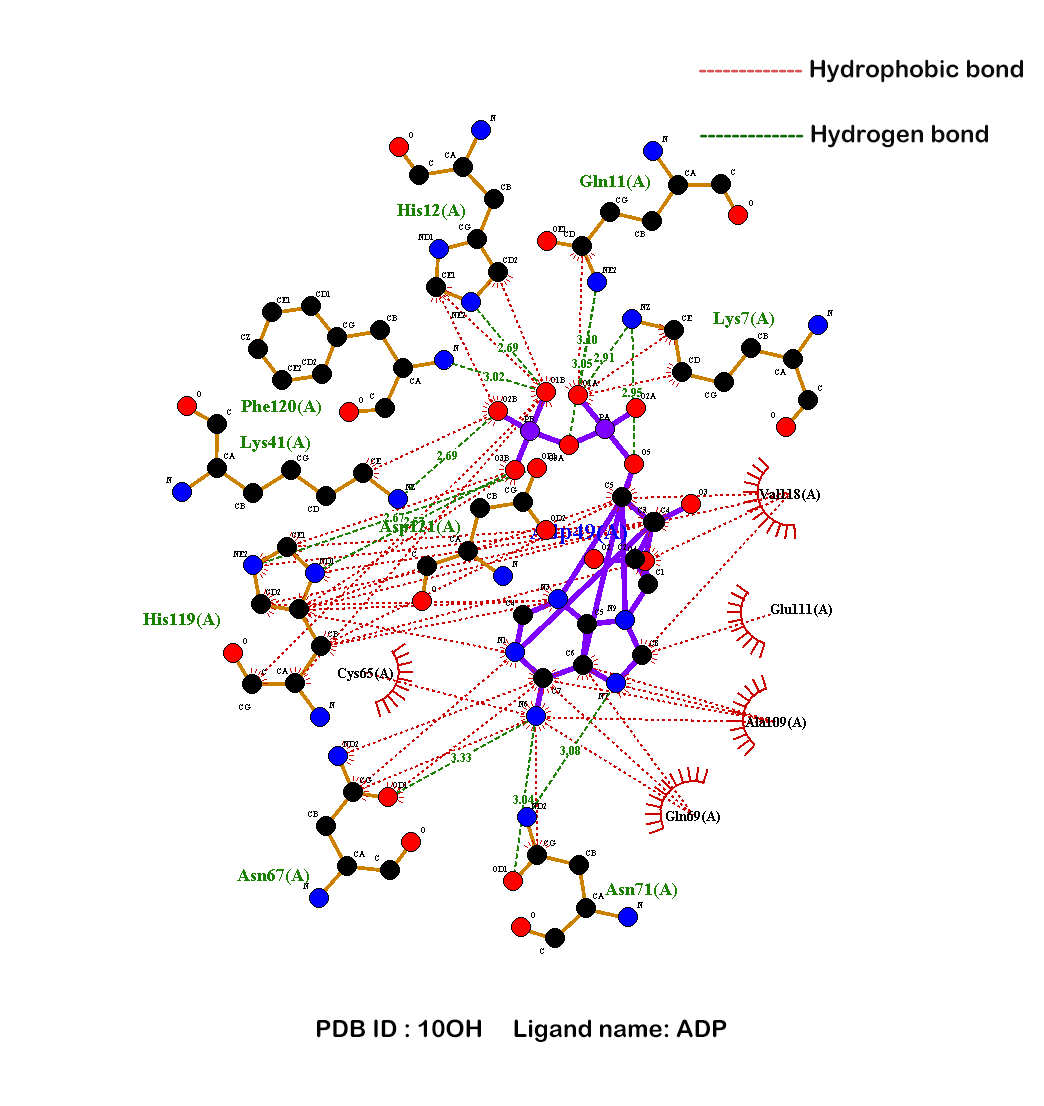
**

**Figure S6.** 2D diagram of the hydrogen bond interactions (indicated by the dotted green lines) and the hydrophobic interactions (indicated by the dotted red lines) between pocket residues and the ligand ADP in complex 10OH visualized by LigPlot+ program.

**
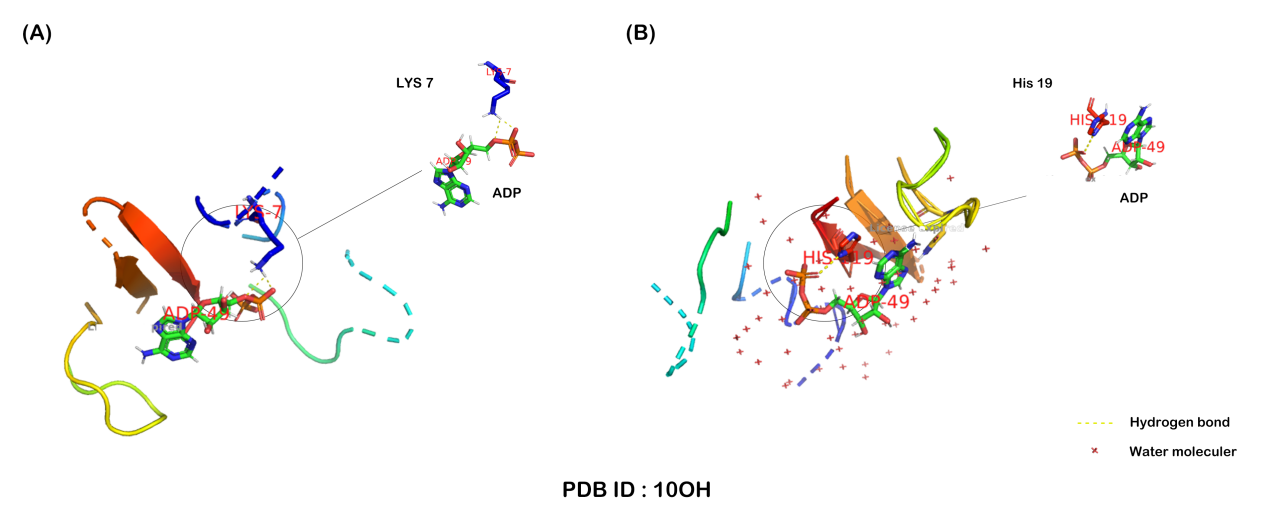
**

**Figure S7.** The 3D structure of the hydrogen interactions between pocket residues Lys7 (A), His19 (B) and the ligand ADP in complex 10OH visualized by PyMol tool.

**
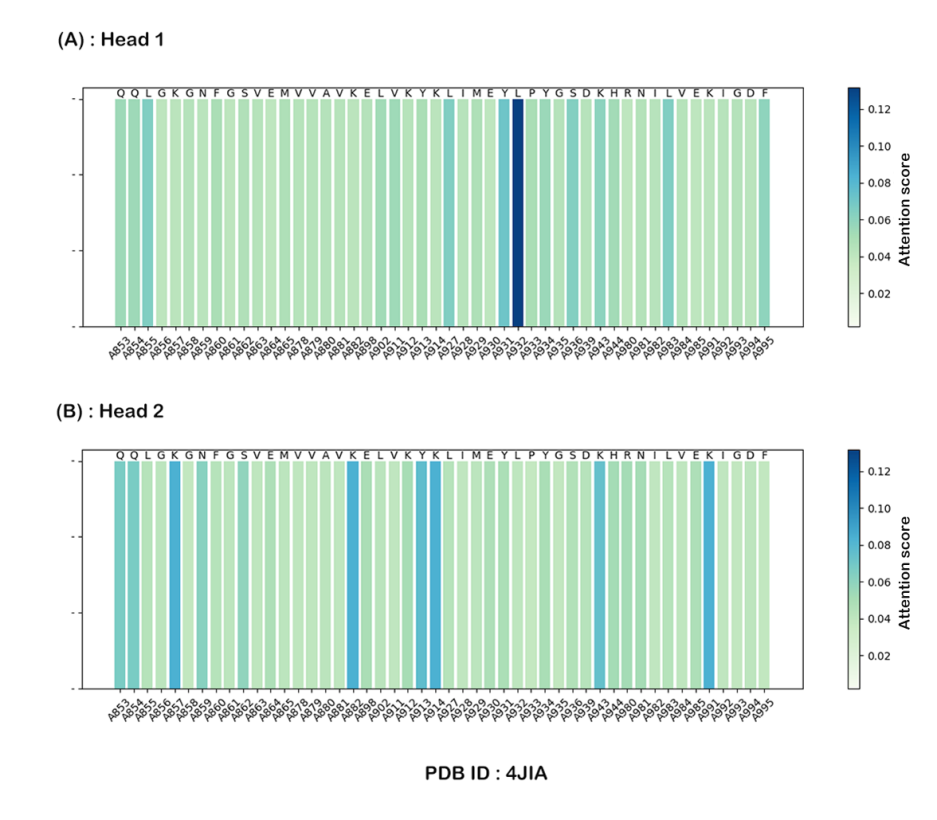
**

**Figure S8.** Visualization of two attention maps of the pocket in complex 4JIA learned by head 1 (A) and head 2 (B) in the cross-attention mechanism.

**
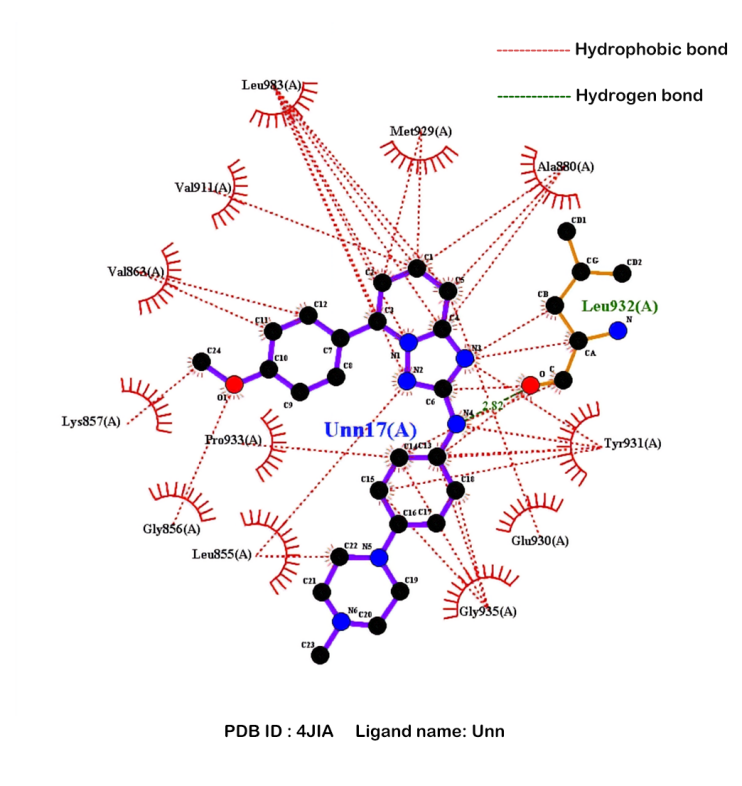
**

**Figure S9.** 2D diagram of the hydrogen bond interactions (indicated by the dotted green lines) and the hydrophobic interactions (indicated by the dotted red lines) between pocket residues and the ligand Unn in complex 4JIA visualized by LigPlot+ program.

**
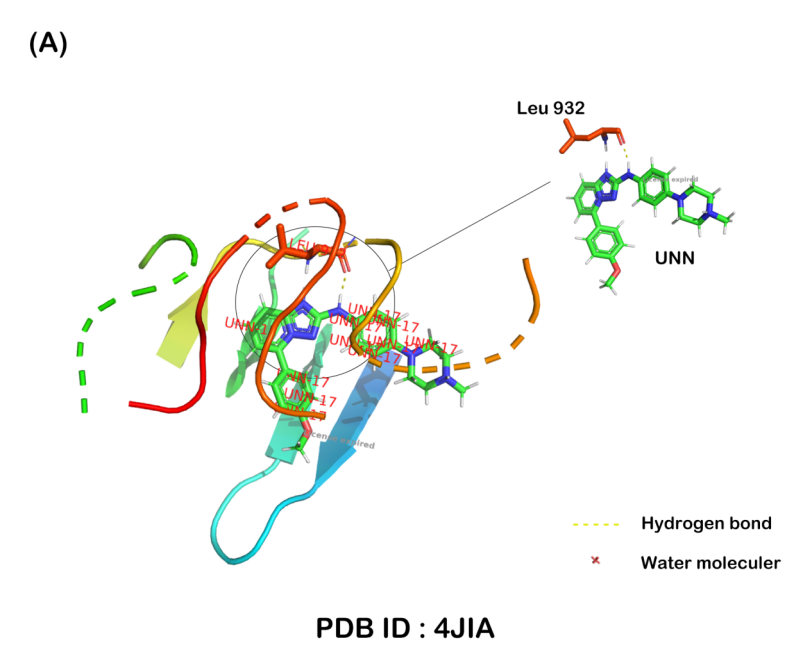
**

**Figure S10.** The 3D structure of the hydrogen interactions between pocket residues (A): Leu932 and the ligand Unn in complex 4JIA visualized by PyMol tool.
